# Moral parochialism and contextual contingency across seven societies

**DOI:** 10.1098/rspb.2015.0907

**Published:** 2015-08-22

**Authors:** Daniel M. T. Fessler, H. Clark Barrett, Martin Kanovsky, Stephen Stich, Colin Holbrook, Joseph Henrich, Alexander H. Bolyanatz, Matthew M. Gervais, Michael Gurven, Geoff Kushnick, Anne C. Pisor, Christopher von Rueden, Stephen Laurence

**Affiliations:** 1 Department of Anthropology and Center for Behavior, Evolution, and Culture, University of California, Los Angeles, CA 90095-1553 USA; 2 Institute of Social Anthropology, FSEV, Comenius University, 820 05 Bratislava 25, Slovakia; 3 Department of Philosophy and Center for Cognitive Science, Rutgers University, New Brunswick, NJ 08901-1107, USA; 4 Department of Psychology, University of British Columbia, Vancouver, British Columbia, Canada V6T 1Z4; 5 Department of Economics, University of British Columbia, Vancouver, British Columbia, Canada V6T 1Z4; 6 Social Sciences Subdivision, College of DuPage, Glen Ellyn, IL 60137-6599, USA; 7 Department of Anthropology, University of California, Santa Barbara, CA 93106-3210, USA; 8 School of Archaeology and Anthropology, The Australian National University, Canberra, Australian Capital Territory 0200, Australia; 9 Jepson School of Leadership Studies, University of Richmond, Richmond, VA 23173, USA; 10 Department of Philosophy and Hang Seng Centre for Cognitive Studies, University of Sheffield, Sheffield S3 7QB, UK

**Keywords:** moral judgement, morality, moral parochialism

## Abstract

Human moral judgement may have evolved to maximize the individual's welfare given parochial culturally constructed moral systems. If so, then moral condemnation should be more severe when transgressions are recent and local, and should be sensitive to the pronouncements of authority figures (who are often arbiters of moral norms), as the fitness pay-offs of moral disapproval will primarily derive from the ramifications of condemning actions that occur within the immediate social arena. Correspondingly, moral transgressions should be viewed as less objectionable if they occur in other places or times, or if local authorities deem them acceptable. These predictions contrast markedly with those derived from prevailing non-evolutionary perspectives on moral judgement. Both classes of theories predict purportedly species-typical patterns, yet to our knowledge, no study to date has investigated moral judgement across a diverse set of societies, including a range of small-scale communities that differ substantially from large highly urbanized nations. We tested these predictions in five small-scale societies and two large-scale societies, finding substantial evidence of moral parochialism and contextual contingency in adults' moral judgements. Results reveal an overarching pattern in which moral condemnation reflects a concern with immediate local considerations, a pattern consistent with a variety of evolutionary accounts of moral judgement.

## 1. Introduction

The propensity to pass sentiment-laden moral judgement on others' actions appears to be a human universal [1]. Negative judgements potentially entail non-trivial costs, as, above and beyond issues of the allocation of time and attention, morally condemning another can bring the actor into conflict with criticized individuals or their allies. Ceteris paribus, individuals who cared little about third parties' actions that did not affect them would have higher fitness than those who embroiled themselves in others' affairs through moral condemnation. At the same time, moral condemnation and attendant moralistic action generate a collective good, as these can play a central role in enhancing cooperation and deterring exploitative behaviour that corrodes the welfare of the group [2]. From the outset of evolutionary biology, morality has thus occupied a central place in efforts to understand both the history of our species and the evolution of cooperation [3].

Though differing in their particulars, a variety of theories indicate that morality is plausibly understood as the product of the conjunction of cultural evolution, which produces norms regulating behaviour, and genetic evolution, which produced psychological mechanisms that increase individual fitness within local culturally constructed social arenas [4–6]. Importantly, despite their differences, all of these evolutionary theories thus predict that third-party moral evaluations will generally be parochial: if moral disapproval ultimately serves to enhance one's reputation in a manner that: (i) deters transgressions against the self, (ii) increases opportunities to participate in mutually beneficial cooperation, and (iii) protects one from higher order punishment [7–11], then, because these benefits will only accrue in the immediate social arena, moral judgements should primarily address recent or ongoing events in one's own social group (the primary exception being the use of moral judgement to elevate the in-group and denigrate an out-group when rival groups conflict—[12]).

With the exception of situations of intergroup conflict—including contexts in which offences against one's group are committed by another group—events that have occurred at a distant place or time will generally have fewer implications for members of one's own group than events that have occurred nearby and recently. As a result, people can be expected to attend less to moral proclamations regarding spatially or temporally distant incidents. Indeed, to the extent that an immediate audience does not have a stake in defending remote targets of disapproval, and such targets will neither learn of the disapproval nor be able to retaliate, such statements, being readily issued, risk being dismissed as cheap talk. Paralleling this, because the condemned actions, being remote, do not disrupt cooperation or coordination within the local social arena, repeatedly voicing such statements may lead others to attend less to the proclaimer's judgements (the problem of ‘crying wolf’ in the moral judgement arena), thereby reducing the individual's ability to amass reputational capital. Relatedly, because an audience's attention is a finite resource, moral condemnation will entail opportunity costs whenever making such pronouncements comes at the expense of other forms of social action. Hence, moral proclamations regarding remote events hold fewer strategic affordances for those who would make them. At the psychological level, when heartfelt, moral condemnation reflects the experience of punitive sentiments that can motivate taking more extensive action against the offender [7], actions that can have great strategic importance for the punisher. Because it is difficult or impossible to take action against remote offenders, there is little value in strongly activating punitive sentiments. Taken together, the above considerations indicate that we can expect natural selection to have refined the input criteria for moral condemnation and the sentiments that underlie and attend it such that remote events will not activate the evolved mechanisms undergirding negative moral evaluation to the same degree as actions that occur in the here and now. This is not to say that actors should assess remote transgressions as acceptable. Rather, remote events should simply trouble actors less than immediate events, evoking weaker sentiments and eliciting less overt condemnation. (The mechanisms at issue afford such muted responses because, to function properly in the local arena, they must produce graded rather than binary outputs as, if punishment is to be administered efficiently, the strength of condemnation must correspond with the seriousness of the transgression.)

Paralleling the above considerations, because local standards change over time, and their application is frequently subject to interpretation, if actors are to accrue individual benefits by passing moral judgement on others' actions, they must be sensitive to indices of current local opinion—moral condemnation can only enhance opportunities for inclusion in cooperative ventures and reduce the likelihood of higher order punishment if the condemner's evaluative criteria match those of her audience. Because authority figures and other high-status individuals are often the arbiters of local norms, people can thus be expected to attend to their pronouncements regarding the moral status of particular actions, as such statements will frequently be determinative of how the community will view these actions. Additionally, the effect of authority figures' opinions on individuals' views will be bolstered by the fact that adamantly adhering to a stance at odds with that of local authorities will often carry real costs. Although formal offices empowering authorities probably first became widespread during the Neolithic Revolution, acephalous hunter–gatherer bands exhibit inequalities in prestige [13], hence we can expect selection to have long shaped the mechanisms responsible for adjusting moral condemnation in light of the opinions of influential individuals.

The above evolutionary perspective diverges sharply from a prominent approach in moral psychology. A voluminous and influential literature, pioneered by Turiel [14], argues that moral rules—putatively rules that address questions of harm, rights or justice—are viewed by adults as inherently both applying to all peoples at all times and being independent of the pronouncements of authority figures. Indeed, such invariance is hypothesized to be a hallmark of moral rules, in contrast to conventions, which are ostensibly recognized by adults as being contingent on local practices and subject to change. This is one version of what we term the theory of universalistic moral evaluation, which holds that the nature of the processes underlying moral assessment are such that, all else being equal, actions that are judged to be immoral are thought to be wrong independent of the time or place in which they occur, and regardless of the opinions of influential or powerful individuals.

Both the evolutionary perspective outlined above and theories of universalistic moral evaluation hold that their respectively predicted features of moral assessment are panhuman. Accordingly, a crucial test for both approaches is a stringent cross-cultural investigation that examines patterns of moral assessment across a diverse range of human societies. Although work on moral judgement has long been conducted cross-culturally (reviewed in [15]), such investigations generally examine members of large-scale societies. While differing from Westerners in many ways, individuals in non-Western large-scale societies are nevertheless likely to be more similar to Westerners with regard to potentially relevant dimensions, such as education and familiarity with formal legal systems, than are members of many small-scale societies [16]. This is further complicated by the fact that much work to date has focused on children's moral development, leaving the key question of panhuman features of adult moral judgements underexplored. To provide a more definitive test of the competing predictions regarding postulated panhuman features of moral judgement, we therefore examined adults' judgements in both large-scale societies and a diverse range of small-scale societies—societies with low population densities where traditional ways of life remain important and which have been influenced to a limited degree by large-scale societies.

Critics of the theory of universalistic moral evaluation have claimed that a significant proportion of adults in the West judge transgressions involving harm, rights or justice to be more acceptable if they occurred long ago or far away, or if the actions were endorsed by authorities. Initial reports in this regard [17] led to debate and further investigations, producing heterogeneous results and no consensus [18–25]. For several reasons, tests to date are inconclusive. First, and most importantly, comparisons across truly dissimilar societies are critically absent. Second, the evolutionary views predict that, because moral disapproval in response to transgressions is shaped by both spatial/temporal distance and the pronouncements of authorities, moral condemnation occupies a graded continuum, contingent on the particulars of each case. However, consonant with the historical focus on children, previous investigations have generally employed simple dichotomous judgements regarding the acceptability of actions, obscuring any such continuum.

Independent of the above debate, several bodies of proximate-level research potentially bear on the predictions at issue. First, in what has been termed the Black Sheep Effect [26,27], both positive and negative social assessments have been shown to be more extreme when applied to in-group members than when applied to out-group members. This bias is consonant with the parochialism predicted by evolutionary theories of morality. The impact of others' actions on both the individual and the group will generally be greater when the actors are members of the in-group, and, correspondingly, the pay-offs to the observer of engaging in social evaluation, be it positive or negative, should be more pronounced in this case [8]; as a consequence, selection can be expected to have shaped mechanisms underlying social evaluation so as to generate more marked praise or condemnation of in-group members relative to out-group members. Consistent with this view, enhanced in-group extremity in moral evaluations and/or the assignation of punishment has been found in the majority of relevant studies, conducted, respectively, with United States, Japanese and German university students ([8,28,29], but see [30]) and maturing British schoolchildren [31]. While relevant to the question at hand, such studies clearly suffer the core limitation of an exclusive focus on large-scale societies.

Whereas research on the Black Sheep Effect and related topics parallels predictions of moral parochialism, a second body of work generates the opposite predictions. Construal Level Theory [32] holds that psychological distance (defined as spatial, temporal, or social distance, or hypotheticality) increases the degree of abstractness with which an event is construed. On this view, moral rules are more abstract than pragmatic considerations, hence more distant events should be construed in more moral terms; as a consequence, more distant transgressions should be judged to be more immoral [33,34]. Although experiments with Israeli and Swedish university students reveal that actions in the distant future are conceptualized in moral terms more than are near-term actions [33–35], these results failed to replicate in a United States university sample [36] and a Serbian university sample [37]; in another United States university sample, the relationship between temporal distance and severity of moral judgement appears to reverse when events in the past are considered (see [38], Experiment 1). Despite these mixed results, given the limited research to date, the predictions of Construal Level Theory, opposite to those of evolutionary theories of moral parochialism, merit testing.

Here, we put the divergent predictions regarding moral judgement to a stringent test: we employ adult samples from five different small-scale societies and two dissimilar large-scale societies; we replace dichotomous judgements of the acceptability of actions with graduated judgements; and we focus on transgressions of important social norms, using scenarios designed to embody the putative hallmarks of moral violations [14].

## 2. Material and methods

We selected small-scale societies that differ with regard to factors central to much cultural variation. Two societies (Tsimane’ and Shuar) are egalitarian indigenous South American groups whose economies are based on horticulture, hunting and fishing; one (Yasawa) is a semi-stratified clan-based indigenous Fijian group reliant on fishing and horticulture; one (Karo Batak) is a clan-based rural Indonesian group focusing on rice agriculture, whereas another (Sursurunga) is a clan-based Melanesian horticulturalist group. Providing points of contrast, data were also collected in Storozhnitsa, a village in western Ukraine, and in relatively affluent urban areas (Santa Monica and San José) in California, USA. (See the electronic supplementary material for details.)

To test the prediction that judgements of the wrongness of transgressions would be contingent on the temporal and spatial locality of the acts and the pronouncements of authority figures, we crafted seven simple vignettes describing clear and substantial harm, violations of rights and/or injustice: a man stealing a stranger's money; a man battering his wife without provocation; a man striking and injuring a friend after the friend unintentionally injured him; a man cheating a stranger in a market transaction; a man knowingly spreading a false rumour that his rival is a thief; the initiator of a fight bribing a witness to lie about who was at fault, resulting in the innocent party being punished; and a man raping an unfamiliar woman (see the electronic supplementary material). For each vignette, after asking the participant to evaluate the given action (‘How good or bad is what [the protagonist] did?’), we then sequentially asked the participant to provide such an evaluation in the event that: (i) a locally appropriate authority figure stated that the action was ‘not bad’; (ii) the action occurred in the distant past; and (iii) the action occurred far away, in another society. Note that, while we anticipated some cross-societal variation in regard to the permissibility of some of these actions, our objective was to test whether those actions viewed in a given society as unequivocally bad would be judged less bad in light of (i), (ii) and (iii), as moral parochialism predicts, or would not be so judged, as both moral universalism and Construal Level Theory predict.

In total, 237 adults across the seven research sites participated (see table 1 for sample characteristics). Vignettes were read aloud in the local language in one of two counterbalanced orders (see the electronic supplementary material). To ensure comprehension, participants were quizzed on each scenario following its presentation; if answers were incorrect, vignettes were re-read, and the process was repeated. Consonant with the simplicity of the vignettes, 96.4% of participants passed the comprehension test at first presentation, with the remainder passing subsequently. Participants were then asked to evaluate each act on a five-point scale (from ‘Extremely Bad’ to ‘Extremely Good’). A printed linear scale (see the electronic supplementary material) was displayed and explained to participants, who pointed to the anchor marks that corresponded with their assessments. To ensure that participants were interpreting the term ‘bad’ as addressing moral concerns (rather than, for example, unfortunate consequences), the same scale was next used to evaluate the effect that the act would have on the protagonist's reputation as a good or bad person. The severity of the transgression was then evaluated in three different contexts, presented in fixed order: authority consent, temporal distance and spatial distance (see the electronic supplementary material).

**Table 1.** Sample characteristics.

		sex ratio	age (in years)	
site	*N*	% female	*M*	s.d.
Tsimane’	30	53.3	37.8	14.39
Shuar	32	62.5	25.9	9.24
Yasawa	49	46.9	41.8	14.77
Karo Batak	34	61.8	35.8	15.75
Sursurunga	30	36.7	43.6	13.75
Storozhnitsa	30	73.3	47.3	15.35
California	32	40.6	28.0	10.04

## 3. Results

Prior to conducting our principal analyses, as an internal validity check, we compared participants' initial ratings of the badness of the seven transgressions with their ratings of the reputational costs suffered by the protagonist (see the electronic supplementary material). Significant positive correlations in all samples indicated that the former indeed reflect moral considerations.

To determine whether participants' judgements of the wrongness of the actions described in the scenarios were affected by the temporal or spatial locality of the acts or the pronouncements of authority figures, we conducted a series of ordinal regressions on participants' judgements, using temporal distance, spatial distance and authority consent as factors (we refer to these variables as TEMPORAL, SPATIAL and AUTHORITY, respectively). For these analyses, we examined only cases in which participants rated the act as ‘bad’ or ‘extremely bad’, as our hypothesis pertains only to judgements of acts judged as immoral [18]. The initial rating of the harmful act thus doubles as a manipulation check to ensure that the participant considered the given act wrong. The vast majority of participants (95.2%, averaged across vignettes) rated acts as ‘bad’ or ‘extremely bad’, and no participant rated more than one act as acceptable, so no participant was excluded from the final aggregate sample (see the electronic supplementary material, table S2, for final sample sizes for each scenario). Using the ordinal package in R [39], we fit a series of cumulative link mixed models (also known as ordered logit models) to the data, using model comparison to select the best-fit of each of the models. The resulting model reveals which of our study variables significantly impacted participants' moral judgements independent of age, sex, education, society and type of scenario evaluated (see the electronic supplementary material for complete analyses and results).

**Figure 1.**
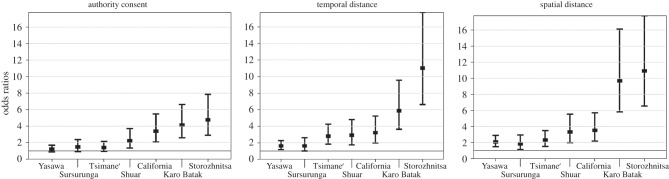
Reductions in the ranked ‘badness’ of transgressions, aggregated across scenarios, as a function of the consent of an authority figure, temporal distance, or spatial distance, presented as odds ratios and their 97.5% confidence intervals. The odds ratios, computed by exponentiating the beta coefficients (*e^*β*^*), provide the odds of a badness judgement falling at a given ranked level or below when the factor is present, relative to when it is absent, across all badness levels. Odds ratios above 1 thus indicate reduced judgements of badness.

If the capacity for moral assessment evolved to operate in variable culturally constructed moral arenas, then such judgements should exhibit lesser condemnation of transgressions removed in time or space, or when the act is condoned by local arbiters of norms. Consistent with this prediction, our best-fit model revealed that the factors TEMPORAL, SPATIAL and AUTHORITY all produced substantial variation in the strength of participants' judgements of the moral wrongness of acts, with an increase in each factor leading to a reduction in wrongness judgements (figure 1; see the electronic supplementary material for details). Participants in all seven societies viewed actions involving gross infliction of harm, violation of rights, and/or injustice as less immoral when they occurred long ago, and the same is true with regard to spatial distance. Endorsement by an authority has this effect in four of the societies sampled, with the other three societies displaying non-significant trends in the direction of reduced severity. These results are robust to differences in sample composition with respect to age, sex and education. Moreover, the patterns emerge despite substantial differences between the samples in the contributions made by the various scenarios. For example, in the Shuar and Storozhnitsa samples, cheating a stranger in the marketplace was a scenario in which judgements were least influenced by spatial or temporal distance or authority consent, whereas the opposite was true in the Yasawa, Tsimane’ and California samples (see the electronic supplementary material for details).

## 4. Discussion

Consistent with the thesis that moral judgements reflect mechanisms that evolved to maximize the benefits derived from assessments of others' behaviour within a circumscribed social arena, across seven very different societies we find evidence that moral judgements of some self-evidently harmful or unjust behaviours are notably parochial and contingent on context. Although participants from the various societies differ in their opinion as to whether a given transgression's wrongness is reduced by spatial or temporal distance or the opinions of authorities, for each society sampled, the overarching pattern across transgressions is clear: there is no evidence of a robust insistence that moral rules are judged to apply equally strongly across such contexts. These results pose a powerful challenge to prevailing views in moral psychology that are committed to the theory of universalistic moral evaluation.

While moral parochialism was evident in each of the societies sampled, nowhere was it absolute—evaluating transgressions that occurred long ago, far away, or were approved of by authority figures generally led participants to view the acts as less bad, but not as perfectly acceptable. A number of possible explanations address this pattern. First, immediate events in the in-group may be the proper domain of biologically evolved mechanisms undergirding moral judgement, such that condemnation of remote events may simply be a partially elicited by-product. Relatedly, because social interconnectedness is a matter of degree, evaluative mechanisms may produce graded judgements in parallel with personal relevance; weak condemnation of remote events may simply reflect the distal end of this continuum. Alternately, while operating at a reduced intensity, biologically evolved mechanisms undergirding moral judgement may apply current local standards to other contexts in order to afford evaluation of out-group members as possible interaction partners and/or maintain the ability to evaluate authority figures' competence. Finally, culturally evolved moral rules prescribing universal applicability may have emerged in the last two millennia in conjunction with technologies allowing for unprecedented travel, communication and conquest, as such rules may leverage these technologies in the service of rapid group expansion.

Everyday conversations suggest that, in many of the societies we sampled, people appear to endorse the universal applicability of moral rules. That our participants' responses nonetheless reveal moral parochialism is consistent with a variety of dual-process psychological models, wherein moral judgements do not result exclusively—perhaps not even primarily—from deliberative moral reasoning, instead being at least partly the product of calculations that occur outside of conscious awareness. Specifically, our results are congruent with dual-process models that stress the importance of emotion [40–42]: given that, in many domains, adaptations shape behaviour through affective influences on motivation and cognition [43,44], such accounts mesh well with our thesis that moral parochialism reflects the central role that dedicated evolved mechanisms play in moral judgement.

The observed reductions in our participants' judgements of the wrongness of acts as a function of spatial or temporal distance are unlikely to merely reflect differences in their interpretation of the questions posed to them. When asked to judge the act in the initial presentation (the present time; a location not far from here), participants can reliably be presumed to be offering their own assessment of the act. However, when asked to judge the act elsewhere, or in the past, might participants have interpreted the question as addressing not their own views, but rather the consensus of people living at the specified place or time? While we cannot exclude this possibility, it does not explain the patterned nature of our findings. It is common to romanticize the past and bemoan present-day moral degeneration [45,46], yet, like spatial distance, temporal distance decreases wrongness judgements in all seven samples, suggesting that perspective-taking probably does not undergird participants' responses. Moreover, whereas a perspective-taking account would predict uniformity in the imagined moral sensibilities of individuals living in remote places or the distant past, within each sample, the scenarios varied substantially in the extent to which spatial and temporal distance inspired reductions in moral condemnation.

The seven societies sampled vary in the degree to which moral judgements are parochial and contingent on the pronouncements of authorities. At one extreme, Ukrainian villagers evince strong reductions in judgements of moral wrongness as a function of temporal distance, spatial distance and authority consent. At the other extreme, Yasawan villagers display much smaller changes in judgement, and do so only in response to temporal and spatial distance. Interestingly, although Western liberal democracies often rhetorically espouse universalist moral positions, urban Californians occupied the middle of the spectrum in this regard. In the future, it will be important to explore which social, psychological or historical factors influence the degree of moral parochialism exhibited in a given society.

Our study of five small-scale societies and two large-scale societies reveals widespread moral parochialism and contextual contingency, suggesting that one or more of the contemporary evolutionary accounts of human morality may well be correct. If so, then, in addition to being both parochial and responsive to authority, moral assessments should be conformist, as individuals frequently prosper by following the views of the majority [47]. History reveals that, together, dependence on the pronouncements of authorities and conformism can undergird genocide and similar horrors, while moral parochialism can undergird indifference to their occurrence elsewhere. Progress in alleviating human suffering may therefore best be achieved by a fuller understanding of the nature and origins of moral judgement.

## Supplementary Material

Description of sites; detailed results; complete methods

## Data accessibility

Data reported in the paper are archived at http://www.philosophy.dept.shef.ac.uk/culture&mind/Data.
