# Supplementary material for: Moral parochialism and contextual contingency across seven societies
Source: Proc Biol Sci. 2015 Aug 22;282(1813):20150907. doi: 10.1098/rspb.2015.0907 (PMC4632614; doi:10.1098/rspb.2015.0907)
Supplement: Description of sites; detailed results; complete methods [file rspb20150907supp1.pdf]

Electronic Supplementary Materials

To Accompany

Fessler et al. *Moral Parochialism and Contextual Contingency Across Seven Societies*

- Descriptions of Research Sites
- Detailed Results
  - Table S1: Correlations between ratings of "badness" and reputational cost across research sites
  - Table S2: Analyzed sample sizes for each of the seven scenarios
  - Complete description of data analyses
    - Data set
    - Data analysis strategy
    - Omnibus analysis
      - Tables S3 and S4: Omnibus models and parameters
    - Fig. S1: Effects of field site
    - Analysis by individual field site, including Tables S5-S18 and Figs. S2-S8
- Additional considerations: Order of presentation of dependent measures
- Protocol modifications by field site
- Complete protocol
- Fig. S9: Scale used to indicate responses
- References

Materials & Methods

Descriptions of Research Sites

Tsimane' — Bolivia — Michael Gurven, Anne Pisor, and Christopher von Rueden, principal researchers

Tsimane' Amerindians live in the central lowlands of Bolivia in the Beni Department, along the Maniqui River and surrounding tributaries. The study was conducted in the village of Ton'tumsi (population: 376). ' is located 27 km from the town of San Borja (population 24,000). The Tsimane' economy is based on slash-and-burn horticulture, fishing, and hunting. They eat plantains, sweet manioc, and rice, supplemented by fish, wild game, and both gathered and planted fruits. Cash cropping of rice is not uncommon in Ton'tumsi, as is purchase of market foods like bread, salt, sugar and dried meat from itinerant merchants and from town. Village unity is weak and the village's elected chief does not wield any substantial power. The social structure is dominated by extended family clusters with a mix of matrilocal and patrilocal residence. Older adults have received little or no schooling. Since 2006, Ton'tumsi has been one of the rare villages to have non-Tsimane' teachers and a secondary school that goes beyond the usual fifth grade (up to eighth grade). Its elementary education has existed for two decades. Traditional Tsimane' religion is animistic, but has syncretized with evangelistic Christianity after the New Tribes Mission established a presence in the 1950s. Belief in sorcery and malevolent spirits is still common. As in other Tsimane villages, Ton'tumsi households lack electricity and plumbing. Exposure to Western media is limited in large part to Christian radio broadcasts (via battery-powered radios) and the action hero movies or soap operas that may be playing in the shops of San Borja.

No direct compensation was offered. However, members of participating communities receive periodic medical care from the investigators, as well as fishhooks, soap, and other amenities given as gifts.

Shuar — Ecuador — H. Clark Barrett, principal researcher

The Shuar are an indigenous Amazonian population in southeastern Ecuador. Participants for this study were recruited from several Shuar villages in the vicinity of Palora, Morona Santiago Province. The participating villages ranged in size from about 100 to 300 inhabitants at the time of the study, and are located near Palora, a town of approximately 6,000. Traditionally, the Shuar practice horticulture, hunting, and fishing, though many adults in this area now participate in wage labor. Staple foods are manioc, plantains, and taro grown in gardens, along with fish and game, but these are now supplemented by store-bought foods. In these villages the main language spoken is Shuar, though all but the elderly are bilingual in Shuar and Spanish (the study was conducted in Spanish). There is a government-sponsored bilingual education system, and most children now receive schooling through high school. All of the participating villages have electricity and some have running water. There is moderate access to Western media in the form of Spanish-language television and DVDs. Many people in this area identify as Christian (either Catholic or Evangelical), though only a small percentage actually attend church services. Traditional beliefs in witchcraft, magic, and healing are still common. There is a strong sense of Shuar identity and culture. In the villages, Shuar music is the most common form of entertainment, and Shuar-language radio the most popular news source.

Participants received $10 in exchange for their participation in a series of studies,

including the present one. At the time, this was approximately equivalent to a day's wage for individuals engaged in market activities.

Yasawa — Fiji — Joseph Henrich and Matthew Gervais, principal researchers

Yasawa Island lies in the northwest corner of the Fijian archipelago, and is about 20 kilometers long and only 1 kilometer wide. The study was conducted in the villages of Teci and Dalomo (combined population approximately 200), which lie about mid-way along the length of Yasawa Island on the windward side. Economically, these communities subsist on a combination of horticulture, littoral gathering, and fishing. Most calories come from cassava and yam, along with bananas and coconuts; market goods such as flour, sugar, rice, and cooking oil, available in small villager-run canteens, contribute to the diet as well. Social and political life is organized by a complex kinship system based on patrilineal clans. In these communities, five clans comprise a *Yavusa*, which is governed by a hereditary chief. Formal education is provided by primary school in Teci, which has roughly 30 students across all grades. Until recently, most people ended their education after finishing primary school, though an increasing number of adolescents have begun attending boarding schools on other islands. Villagers have few books, except for the Bible, and no access to broadcast television. The researchers do occasionally show American movies on their laptops, and a few households have television sets that are used to watch recorded rugby matches. Religion in these villages is a syncretic mix of Christianity, including both Methodist and Assemblies of God Churches, as well as ancestor beliefs, and some witchcraft. More details on these villages can be found in the supplemental materials of [1].

No compensation was offered; research has been conducted at this site for many

years, and community members are periodically compensated for participation in a wide variety of studies.

Karo Batak — Indonesia — Geoff Kushnick, principal researcher

The traditional homelands of the Karo Batak comprise both high and lowland areas of periequatorial North Sumatra, Indonesia. Research was conducted in two villages, Doulu (1,003 residents) and Laubuluh (791 residents). The villages are relatively ethnically homogeneous. Almost everybody in both villages practices a mix of subsistence and cash-crop agriculture, with some people supplementing their incomes as teachers, civil servants, drivers, and shopkeepers. In Doulu, wet-rice agriculture predominates, along with the growing of vegetables and fruits. In Laubuluh, dry-rice agriculture predominates, again with a mix of vegetable and fruit cultivation. Karo Batak society centers around five exogamous clans (*marga*). Marriage, which is accompanied by the payment of a bridewealth, creates very specific social obligations between wife-giving (*kalimbubu*) and wife-taking (*anakberu*) families. Over the past 150 years, missionary, colonial, and national influences have changed Karo Batak lifestyles. For instance, although they practiced a traditional, animistic religion in the past, today the Karo Batak are primarily Protestant or Catholic. Both villages have a primary school; most adults have at least some junior high school education, while a quarter of the residents have some senior high school, and a small minority have some college education. There is some exposure to Western media, in the form of music and movies, but less than a third of the households have a television.

Participants were paid 15,000 rupiah (approximately $2.00); typical daily incomes in the region ranged between 50,000 and 100,000 Rp.

Sursurunga — Papua New Guinea — Alexander Bolyanatz, principal researcher

The east coast of southern New Ireland is home to most of the 4000 speakers of Sursurunga, an Austronesian language. Sursurunga territory is spread across nineteen nucleated settlements spanning 70 kilometers of coastline and includes the primary research sites of Tekedan (population 130), Himaul (population 110), and Nokon (population 235) villages, which are situated in the middle of that 70 kilometer stretch. Most inhabitants of these villages rely primarily on horticulture, with tubers such as sweet potato, yams, and taro being grown in swidden gardens. Cacao and copra holdings provide some cash. Matrilineal descent regulates inheritance and the sequence of mortuary feasts; matrilineal moieties are exogamous. The median number of years of formal schooling is six, and the mean is slightly above that. Indigenous beliefs (especially those related to illness, healing, and gardening) are common, although the United Church (an amalgam of Methodism and Congregationalism) is the dominant religious institution in these three villages, and there is a small but growing Pentecostal movement in the area. Villagers have few books except for religious (Christian) materials, most of which are in the local language. At the time that research was conducted, there was no cell phone reception in the area, nor any way to connect to the Internet. The only electricity available was produced by generators. On occasion, these generators were used for all-night video/DVD showings for fundraising purposes. Action films and sports were the most commonly viewed presentations. See [2] for a more thorough description of the research area.

No compensation was offered for participation; rather, the investigator, who has worked at this site for many years, periodically provides both personal and community-wide assistance, including medical care, unrelated to participation.

Storozhnitsa — Ukraine — Martin Kanovsky, principal researcher

The village of Storozhnitsa is located in the district Transcarpathia (Western Ukraine) in the mid-southern part of the Carpathia Mountains. The village consists of some 2,500 people, ethnically composed of roughly 61% Ukrainians, 22% Slovaks, 15% Hungarians, and a smattering of Romani (Gypsies), Russians, and Rusyns (Ruthenians). Ethnically mixed marriages are not unusual, and the vast majority of inhabitants are bi- or trilingual. Traditionally, most inhabitants are farmers, although recently increasing numbers work in services and small business. Even people who are employed by the State (teachers, officials, etc.) are dependent on the land as an additional source of food to keep and/or to sell on the market, as their salaries are paid irregularly. Due to the border location of the village (Slovakia and Hungary are very close), its inhabitants profit from small inter-border trade. In the last decade people have begun moving into the village from the nearby district capital of Uzhgorod (12 km away), and the village has begun to take on a somewhat suburban character, but its mostly rural infrastructure (roads, water supply, sanitation) is nonetheless primitive. There is electricity in the village, but its supply is far from reliable. There is an elementary school (9 grades) in the village, and literacy is close to 100%. Children generally attend secondary educational institutions in Uzhgorod. Beyond that, some families send their children to Hungary or Slovakia for additional education. Approximately 60% of residents are Greek-Catholics, 25% are Roman Catholics, and the rest belong to (Calvinist)

Protestantism and the Eastern Orthodox Church. In the village, people are able to receive various TV signals, and they regularly watch Ukrainian, Slovak, and Hungarian broadcasting. However, most information presented in these media is concentrated mainly on national and sometimes local politics, and the coverage of world events is very limited except for very short highlights.

Participants were paid 10 Ukrainian Hryvnia (UAH) (approximately $1.35 U.S. at the time); the average daily income was 40 UAH, albeit with substantial variance. Data were collected several years prior to the civil war currently afflicting Ukraine.

Urban California – U.S.A. – Daniel Fessler and Colin Holbrook, principal researchers

Participants were recruited in bookstores and coffee shops in two relatively affluent large urban areas, Santa Monica and San José; interviews were conducted on site. Participants were paid $10 for their participation.

Detailed Results

Validity Check

Participants were asked to rate "How good or bad" the actions of the protagonists were. Such assessments can reflect considerations, such as pragmatic consequences, that are independent of moral considerations. To measure whether participants were providing ratings that were grounded (at least in part) in moral evaluations, participants were also asked to rate the reputational consequences of the actions for the protagonists. As an elementary check of the validity of our procedure, we then compared these two types of ratings. Analyzed across research sites, participants' initial ratings of the badness of the seven transgressions were internally reliable ($\alpha = .74$). Each participant's ratings were averaged across transgressions for a composite score. Ratings of the severity of the reputational costs that a protagonist would incur should others learn that he had committed the transgressions were also reliable ($\alpha = .73$), and were similarly averaged to create a composite reputation cost score. The composite transgression badness and reputational cost scores were then correlated for each sample (see Table S1, below). As predicted, the two scores were significantly correlated in all samples, indicating that, as we intended, moral considerations underlaid participants' assessments of the acts as bad.

Table S1. Correlations between ratings of “badness” and reputational cost across research sites

	N	*r*	*p*
Tsimane’	30	.82	.001
Shuar	32	.61	.001
Yasawa	49	.37	.010
Karo Batak	34	.38	.025
Sursurunga	30	.66	.001
Storozhnitsa	30	.57	.001
California	32	.62	.001
Combined	237	.57	.001

Table S2. Analyzed sample sizes for each of the seven scenarios

	Stealing	*Wife Battery*	*Violence Following Accidental Harm*	*Marketplace Cheating*	*Defamation*	*Unjust Perjury*	*Rape*
Tsimane’	28	29	26	21	26	25	25
Shuar	32	32	32	32	31	32	--
Yasawa	47	43	45	48	46	48	48
Karo Batak	28	33	30	34	34	34	31
Sursurunga	30	30	27	30	29	30	29
Storozhnitsa	30	30	24	30	30	30	25
California	32	32	28	32	31	32	31
**Combined**	227	229	212	227	227	231	189

Note. Samples are composed of those individuals who rated the given act as “bad” or “extremely bad” at baseline (i.e., in the present time and place, without mention of the opinions of an authority figure).

Data Analyses

To analyze effects of our variables of interest on subjects' judgments, following Christensen [3], we conducted a series of ordinal regressions using the `ordinal` package in R, version 3.0.2 [4]. In each case we used cumulative link models (CLM) or cumulative link mixed models (CLMM) to estimate the impact of the different factors in our study design, including our scenario variables, on subjects' judgments. We conducted both omnibus analyses on the entire dataset (summarized in the main text) and separate analyses for each study population. Here we describe the results of each analysis in detail.

<u>Data Set</u>

Our data set included the following variables (the full dataset is available online at http://www.philosophy.dept.shef.ac.uk/culture&mind/Data/)**.** Each subject was identified by a unique identification number (I.D.) and each subject's record included the demographic variables SEX, AGE, and EDUCATION (in years). The variable FIELDSITE contained a code for each of our seven field sites, and was treated as a random factor in the analyses below. Two hundred and thirty-seven participants from seven field sites (Tsimane', Shuar, Karo Batak, Sursurunga, Yasawa, Storozhnitsa, and California) each assessed seven different scenarios in each of the three treatment conditions, as well as the baseline.

Each of our seven individual scenarios (e.g., "Stealing," "Wife battery") was coded as the random factor STORY. There were three fixed factors corresponding to the systematic variations in authority, temporal distance, and spatial distance in our scenarios: AUTHORITY, TEMPORAL, and SPATIAL, respectively. Each of these was coded 0 for absence of the factor and 1 for presence, and all were treated as fixed factors in the following analyses. The dependent variable, RESPONSE, was the badness judgment given by the

participant in each case (negative values of RESPONSE indicate "bad" judgments, and positive values indicate "good" judgments, such that -2 = "extremely bad" and +2 = "extremely good"). Note that only cases in which badness responses were at least "bad" or "extremely bad" were included in the analyses reported below, as our hypotheses concerned judgments of cases deemed to be morally bad [5]. Averaging across research sites and scenarios, only 4.8% of transgression judgments were dropped per this criterion; no individual participant was dropped from more than one scenario, hence the final sample, aggregated across scenarios, contains all participants (see Table S2 for final sample sizes for each scenario). In the majority of cases wherein altering authority consent, temporal distance, or spatial distance affected participants' responses, changes from the participant's baseline assessment were on the order of one or two points in the five-point scale; however, there were instances in which subsequent ratings went as high as +2, "extremely good".

Thus, our study design consisted of three fixed factors, AUTHORITY, TEMPORAL, and SPATIAL. We predicted that each of these three factors – authority consent, temporal distance, and spatial distance – would account for variation in subjects' badness judgments, with an increase in each factor decreasing the severity of the judgments (i.e., increasing the perceived acceptability of the given action). We assessed these predictions by examining the effects of AUTHORITY, TEMPORAL, and SPATIAL on the RESPONSE variable in our regressions. In addition, we examined effects of the various random factors described above, including FIELDSITE, on the variable RESPONSE.

Data Analysis Strategy

We conducted two types of analysis: first, a series of omnibus analyses on the data from

all of our field sites, for cases in which participants provided an initial judgment of "bad" or "extremely bad." These are reported in the section titled Omnibus Analyses. Second, we conducted separate analyses of the data from each field site, individually. These field site-specific analyses follow the section describing the omnibus analyses. An overview of the results of the omnibus analyses are described in the main text of the paper; we provide the field site-specific analyses here as additional material.

All of our analyses used model comparison techniques in order to produce the statistical model most consistent with the data. This procedure involves constructing statistical models that progressively include more of our measured variables, and observing whether or not including these variables increases the fit of the model. For a variable to increase the fit of the model, it must explain significantly more of the variance in the dependent measure, RESPONSE, than models without that variable included. We used two statistical techniques to compare models. The Akaike Information Criterion, or AIC, uses a statistical formula to trade off increased model fit, a general consequence of adding more variables, against the loss in parsimony produced by adding additional variables. Likelihood Ratio Tests, or LRT, compare the fit of contrasting models using a null hypothesis approach, generating *p* values which decrease as a function of differences in model fit. For our data these approaches produced converging answers, hence, for reasons of space, we do not report both for every model.

CLM and CLMM are types of regression that assume that the dependent variable is ordinal, or ranked in nature (i.e., they are not a scalar measure of a quantity). In each case we fitted a minimal baseline model that did not include any of our independent variables; this only modeled the intercept of the variable RESPONSE. We then compared this to a model

including the three manipulated variables designed to test our predictions: AUTHORITY, TEMPORAL, and SPATIAL. Following this, we added additional variables, in order, and compared the fits of the resulting models to arrive at the best-fit model.

CLM models take the following form, using the example of a CLM modeling the effects of AUTHORITY, TEMPORAL, and SPATIAL on the variable RESPONSE:

$$\text{logit}(P(Y_i \leq j)) = \theta_j - \beta_1(\text{AUTHORITY}_i) - \beta_2(\text{TEMPORAL}_i) - \beta_3(\text{SPATIAL}_i)$$

This function models the cumulative probability that the *i*th rating will fall in the *j*th category or lower, where i = 1 to 6168 are the observations of the variable RESPONSE, and j = 1 to 5 are our ordinal response categories of badness (-2 to +2). Thus, the CLM enables us to ask how variation in our fixed factors AUTHORITY, TEMPORAL, and SPATIAL result in variation in participant judgments, while treating the value of those judgments as rank-ordered, rather than magnitudes. CLMM models include, in addition to fixed parameters, estimates of random effects, for example:

$$\text{logit}(P(YI \leq j)) = \theta_j - \beta_1(\text{AUTHORITY}_i) - \beta_2(\text{TEMPORAL}_i) - \beta_3(\text{SPATIAL}_i) - u_1(\text{ID}_i) - u_2(\text{Story}_i) - u_3(\text{FieldSite}_i)$$

Thus, CLMs are used in cases where we model only fixed effects, while CLMMs are used when random effects are included.

Omnibus Analyses

In our omnibus analyses we fitted a series of CLM models to the RESPONSE data from all the field sites, for cases in which scenarios were judged “bad” or “extremely bad.” We fitted a total of eight models, including the baseline model with no predictor variables included (i.e., modeling intercept of RESPONSE only). Following the baseline model, we

added AUTHORITY, TEMPORAL, and SPATIAL as fixed effects, followed by FIELDSITE, STORY, and subject I.D. (to model between-subject variation in RESPONSE judgments) as random effects, and then added the additional fixed effects of EDUCATION, SEX, and AGE. The results of these regressions, including the estimated parameter values of each variable and the AIC statistics for each model, are summarized in Table S3.

Table S3. Summary of omnibus models

	Models							
	0 (Baseline)	1	2	3	4	5 (Best fit)	6	7
**Fixed effects** β (standard error), Wald *p* value†								
AUTHORITY	--	.45 (.07)***	.49(.07)***	.50(.07)***	.70(.08)***	.69(.08)***	.70(.08)***	.69(.08)***
TEMPORAL	--	.70(.07)***	.76(.07)***	.78(.07)***	1.03(.08)***	1.03(.08)***	1.03(.08)***	1.03(.08)***
SPATIAL	--	.77(.07)***	.83(.07)***	.85(.07)***	1.14(.08)***	1.14(.08)***	1.14(.08)***	1.14(.08)***
EDUCATION	--	--	--	--	--	-.07(.03)*	-.07(.03)*	-.08(.03)*
SEX	--	--	--	--	--	--	.06(.19)	.06(.19)
AGE	--	--	--	--	--	--	--	-.003(.007)
**Random effects** Variance estimate (standard deviation)								
FIELDSITE	--	--	.30(.55)	.14(.37)	.56(.75)	.52(.72)	.52(.72)	.53(.73)
STORY	--	--	--	.34(.58)	.22(.47)	.22(.47)	.22(.47)	.22(.47)
I.D.	--	--	--	--	1.91(1.38)	1.86(1.36)	1.86(1.36)	1.86(1.36)
**AIC value**	12001	11856	11390	11243	9975	9971	9973	9975

† *p* value codes: *** < 0.001, ** < 0.01, * < 0.05 (note that *p* values do not apply for random effects); -- variable not present in model

Inspection of the AIC values reveals that the model with the lowest AIC value – corresponding to the best-fit model – was Model 5, which included AUTHORITY, TEMPORAL, SPATIAL, FIELDSITE, STORY, I.D., and EDUCATION. Table S4 summarizes the parameter estimates of this model in more detail.

Table S4. Parameters of best-fit omnibus CLMM model (Model 5)

Fixed effects							
Factor	β (Maximum likelihood estimate)	95% CI	Std error	*z* value	Wald *p* value	LRT using single-term deletions (with *p* values)	AIC for single-term deletions (Whole model = 9971)
AUTHORITY	.69	.53 – .85	.08	8.5	<.001	73.8 (<.001)	10043
TEMPORAL	1.03	.87 – 1.19	.08	12.8	<.001	166.7 (<.001)	10136
SPATIAL	1.14	.98 – 1.30	.08	14.0	<.001	202.1 (<.001)	10171
EDUCATION	-.07	-.13 – -.01	.03	-2.4	.02	5.6 (.02)	9975
Random effects							
Factor	Estimated variance	Standard deviation					
FIELDSITE	.52	.72					
STORY	.22	.47					
I.D.	1.86	1.36					

The positive β coefficients for each of the fixed factors AUTHORITY, TEMPORAL, and SPATIAL reveal that these factors had significant positive effects on participants' responses, where more positive responses reflect judgments of less badness; thus, each factor significantly reduced participants' badness judgments, globally. The *p* values in this table are Wald-based *p* values that estimate probabilities that the β parameters are zero. The 95% confidence intervals

are estimated based on the profile likelihood function, which allow asymmetric confidence intervals, and are more accurate than Wald-based confidence intervals, which are based on standard errors.

To confirm the individual contribution of each fixed factor, we performed both likelihood ratio tests (LRT) and computed AIC values using the single-term deletion method, in which each factor was removed from the full model and the resulting changes in model fit were assessed. As shown in Table S4, both LRT and AIC revealed that each of the factors AUTHORITY, TEMPORAL, and SPATIAL had large effects on model fit, each one significantly reducing fit when removed. In addition, EDUCATION had a small effect, reaching $p < .05$ in the LRT.

Thus, our best-fit omnibus model confirmed our primary prediction: namely, that each of the factors AUTHORITY, TEMPORAL, and SPATIAL would significantly reduce participants' badness judgments.

Effects of STORY varied across field site, and these effects will be investigated in greater detail below. In Figure S1, below, we plot the effects of FIELDSITE using conditional modes (the modes of the distributions for the random effects given the estimated parameters of the model and the observed data), with 95% confidence intervals based on the conditional variance.

Figure S1. Effects of FIELDSITE on participant responses (pooled across all scenarios, combining baseline, authority, temporal, and spatial conditions for each scenario)

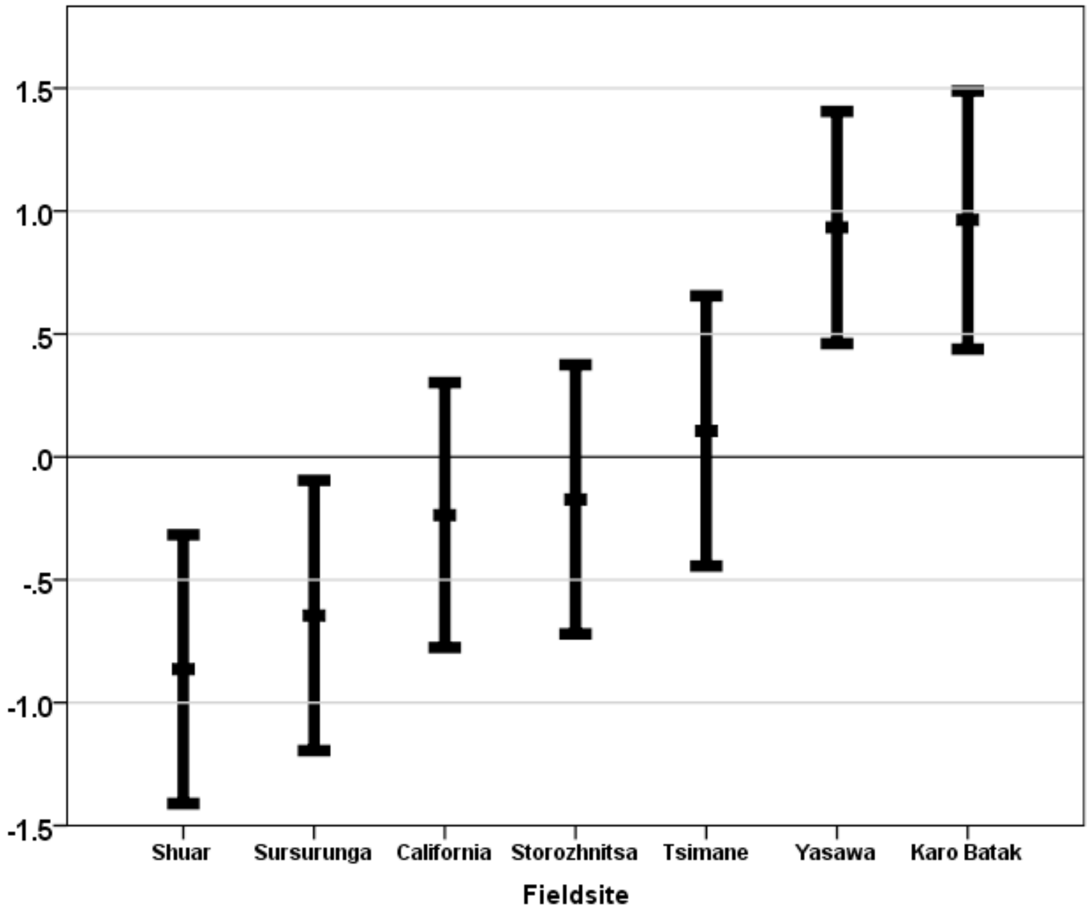

Participants in the Shuar and Sursurunga samples gave the lowest (i.e., most “bad”) ratings of the actions recounted in the scenarios, followed by California, Storozhnitsa, and Tsimane’, while participants in the Yasawa and Karo Batak samples gave the highest ratings of the acceptability of these actions. The significant FIELDSITE effect indicates that participants in different societies either a) differ in their assessments of the degree of moral wrongness of the transgressions in aggregate, or b) differ in their use of the various components of the 5-point scale using which responses were reported.

Analysis By Individual Field Site

To examine effects of our story variables and the effects of age, sex, and education on participants' responses within field sites, we fitted individual CLM models to the data from each field site individually. For each site we constructed a baseline model with AUTHORITY, TEMPORAL, and SPATIAL as fixed effects and individual I.D. as a random effect, and then added additional variables (STORY, SEX, EDUCATION, and AGE) one at a time, assessing differences in model fit using likelihood ratio tests in order to arrive at the best-fit model. Below we report the results of these models by field site.

Tsimane'

The best-fit model for the Tsimane' included the fixed factors AUTHORITY, TEMPORAL, and SPATIAL, and the random factors I.D. and STORY. A summary of this model is shown in Table S5.

Table S5. Tsimane' best-fit model: AUTHORITY, TEMPORAL, SPATIAL, I.D., and STORY

Fixed effects						
Factor	β (Maximum likelihood estimate)	95% CI	Standard error	*z* value	Wald *p* value	LRT using single-term deletions (with p values)
AUTHORITY	.344	-.069 - .756	.210	1.63	.103	2.67 (.102)
TEMPORAL	1.03	.615 – 1.45	.213	4.85	<.0001	24.0 (<.0001)
SPATIAL	.836	.422 – 1.25	.211	3.96	<.0001	15.9 (<.0001)
Random effects						
Factor	Variance estimate	Standard Deviation				
I.D.	.838	.916				
STORY	.077	.278				

A likelihood ratio test, as well as AIC statistics, indicated that STORY significantly improved fit compared to the baseline model without it (LR statistic = 5.61, p = .018). Similarly, a model that included I.D. had significantly better fit than a model with AUTHORITY, TEMPORAL, and SPATIAL alone (LR statistic = 67.7, p < .0001).

Likelihood ratio tests using single-term deletion (i.e., removing one factor while holding others constant) indicated that among the Tsimane', the TEMPORAL and SPATIAL manipulations had significant effects on badness judgments, but AUTHORITY did not. However, the AIC statistic for the model increased slightly when AUTHORITY was removed, so we include it in the best-fit model.

Using the same method, we individually added SEX, EDUCATION, and AGE as fixed factors to the best-fit model, and none of these improved model fit (SEX LR statistic = 2.42, p = .12; EDUCATION LR statistic = .157, p = .69; AGE LR statistic = .192, p = .66). Therefore the best-fit model includes AUTHORITY, TEMPORAL, SPATIAL, I.D., and STORY.

Table S6 summarizes the parameters of models with different parameters included or excluded.

Table S6. Comparison of models for Tsimane' data

Factor	Baseline β values (sig.)	Model2 β values (sig.)	Model3 β values (sig.)	Model4 β values (sig.)
AUTHORITY	0.34 *ns* (p < 0.11)	0.34 *ns* (p < 0.11)	0.34 *ns* (p < 0.11)	0.34 *ns* (p < 0.11)
TEMPORAL	1.03 ***	1.03 ***	1.03 ***	1.03 ***
SPATIAL	0.84 ***	0.84 ***	0.84 ***	0.84 ***
Sex	NA	*ns* (p < 0.12)	NA	NA

Education	NA	NA	*ns* (p < 0.70)	NA
Age	NA	NA	NA	*ns* (p < 0.67)
Overall fit of the model (-2LL)	NA	*ns* (p < 0.12)	*ns* (p < 0.70)	*ns* (p < 0.67)

*** < 0.001, ** < 0.01, * < 0.05, *ns* non-significant, NA non-applicable

The effects of STORY among the Tsimane' are shown graphically in Figure S2, which illustrates STORY effects via conditional modes with 95% confidence intervals based on the conditional variance (we use conditional modes, as above, because STORY effects are not parameters).

Figure S2. Effects of STORY in the Tsimane' Sample

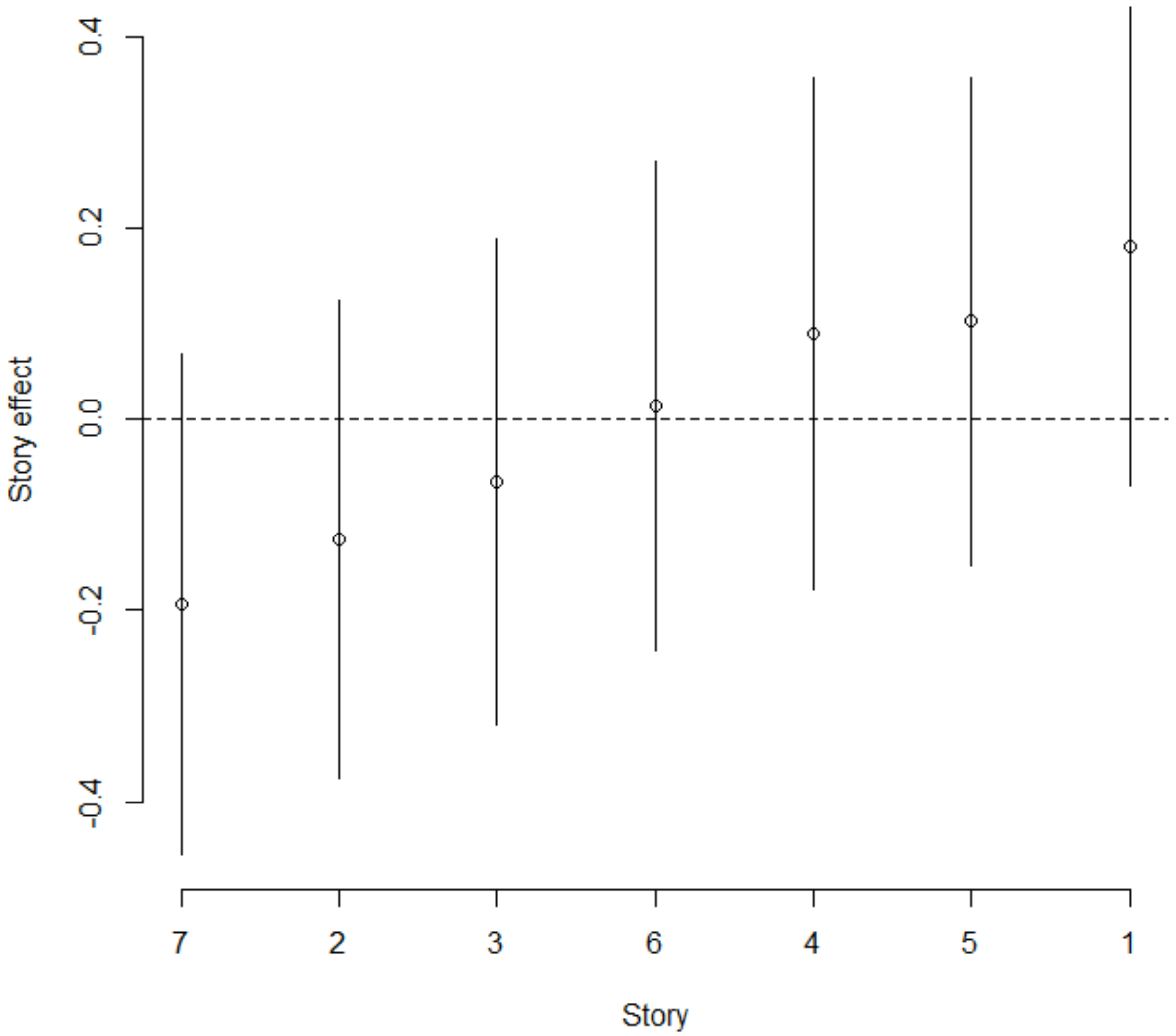

We see that STORY effects are small for Tsimane', meaning that changes in the assessed "badness" of the transgression as a function of temporal or spatial distance or authority consent is not highly dependent on the nature of the transgression. Scenario 7 (Rape) and Scenario 2 (Wife Battery) are the least responsive to such changes in context, while Scenario 1 (Stealing) is the most responsive in this regard. However, overall, the effect of STORY is small.

Shuar

The best-fit model for the Shuar included the fixed factors AUTHORITY, TEMPORAL, and SPATIAL, EDUCATION[1], and AGE, and the random factors I.D. and STORY. A summary of this model is shown in Table S7.

Table S7. Shuar best-fit model: AUTHORITY, TEMPORAL, SPATIAL, EDUCATION, AGE, I.D., and STORY

Fixed effects					
Factor	β (Maximum likelihood estimate)	95% CI	Standard error	*z* value	Wald *p* value
AUTHORITY	.803	.297 – 1.31	.258	3.11	.0019
TEMPORAL	1.07	.558 – 1.57	.259	4.11	<.001
SPATIAL	1.20	.690 – 1.71	.259	4.63	<.0001
EDUCATION	-.215	-.423 – -.0063	.106	-2.02	.043
AGE	-.0932	-.188 – .0014	.0483	-1.93	.054
Random effects					
Factor	Variance estimate	Standard Deviation			
I.D.	4.39	2.09			
STORY	.378	.615			

As above, we first fit a baseline model with variables AUTHORITY, TEMPORAL, SPATIAL, then added I.D., which significantly improved the fit of the model (LR statistic = 307.6, $p < .0001$). In addition, single-term deletions revealed all three fixed effects, AUTHORITY, TEMPORAL, and SPATIAL, to be significant (AUTHORITY: LR statistic = 8.79, $p = .003$; TEMPORAL: LR statistic = 15.4, $p < .0001$; SPATIAL: LR statistic = 20.0, p <

[1] Data on level of education achieved were missing for 10 out of 32 participants. For the purposes of analysis, for these individuals, we employed the mean number of years of education (7.64) found in the remainder of the sample.

.001). Thus, there was significant variation in responses between individuals, as well as significant effects of each of our manipulated scenario variables.

We next added STORY, which significantly improved the fit of the model (LR statistic = 32.5, $p < .0001$). Adding SEX did not improve the model fit (LR statistic = 1.35, $p = .245$), but adding EDUCATION did (LR statistic = 4.42, $p = .035$), as did adding AGE (LR statistic = 4.16, $p = .042$). Thus, the final best-fit model included the fixed factors AUTHORITY, TEMPORAL, and SPATIAL, EDUCATION, and AGE, and the random factors I.D. and STORY. Table S8 summarizes the parameter estimates of the models described above.

Table S8. Comparison of models for Shuar data

Factor	Baseline β values (sig.)	Model2 β values (sig.)	Model3 β values (sig.)	Model4 β values (sig.)
AUTHORITY	0.80 ***	0.80 ***	0.80 ***	0.80 ***
TEMPORAL	1.07 ***	1.07 ***	1.07 ***	1.07 ***
SPATIAL	1.20 ***	1.20 ***	1.20 ***	1.20 ***
Sex	NA	*ns* (p < 0.25)	NA	NA
Education	NA	NA	- 0.25 *	- 0.22 *
Age	NA	NA	NA	- 0.09 *
Overall fit of the model (-2LL)	NA	*ns* (p < 0.25)	*	*

*** < 0.001, ** < 0.01, * < 0.05, *ns* non-significant, NA non-applicable

Figure S3 illustrates STORY effects via conditional modes with 95% confidence intervals based on the conditional variance:

Figure S3. Effects of STORY in the Shuar Sample

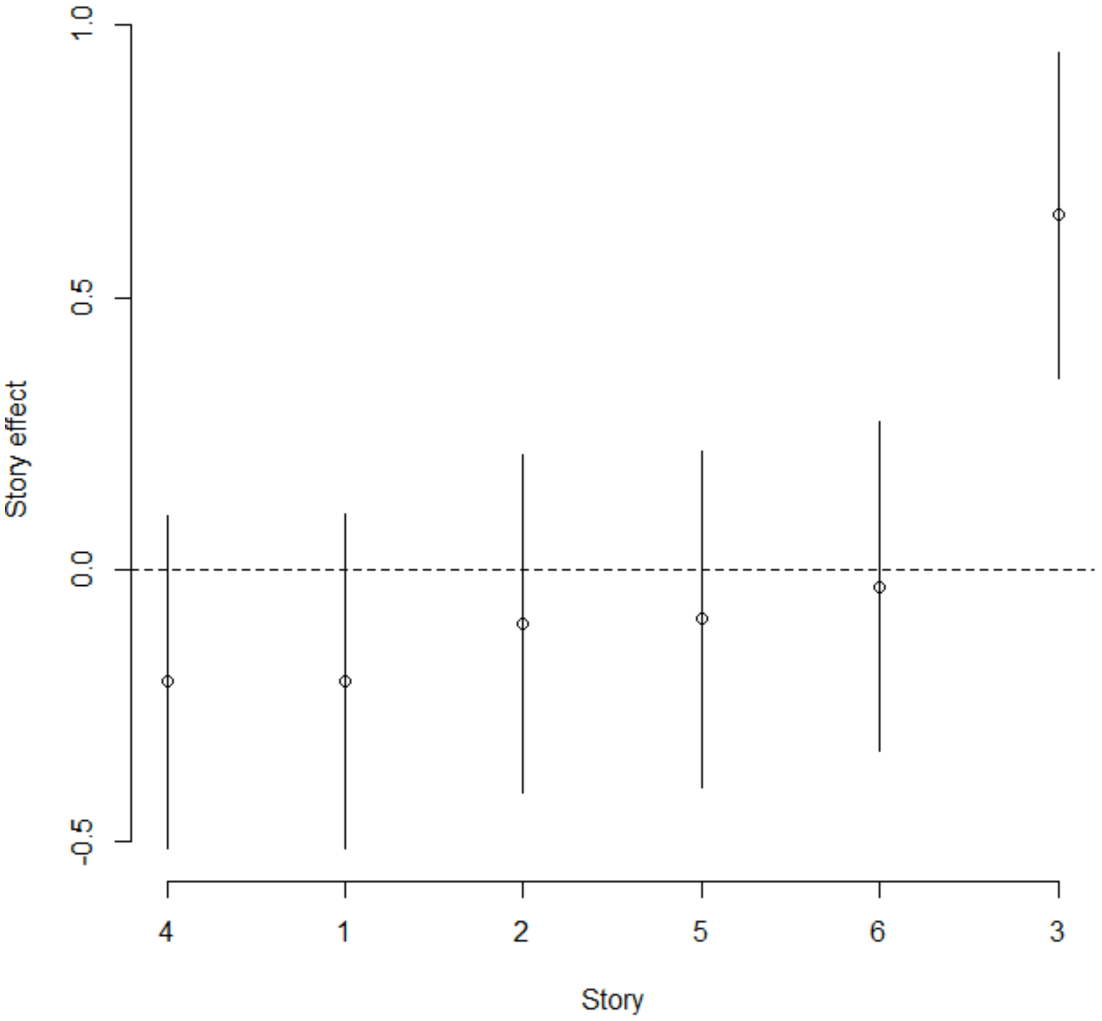

In the Shuar sample, Scenario 4 (Marketplace Cheating), Scenario 1 (Stealing), Scenario 2 (Wife Battery), Scenario 5 (Defamation), and Scenario 6 (Unjust Perjury) all had only small effects, meaning that participants' evaluations were not substantially influenced by temporal or spatial distance or authority consent; in this sample, only evaluations of Scenario 3 (Violence Following Accidental Harm) display marked reductions in "badness" ratings as a function of such contextual changes.

Yasawa

The best-fit model for Yasawa included the fixed factors AUTHORITY, TEMPORAL, and SPATIAL, and the random factors I.D. and STORY. A summary of this model is shown in Table S9.

Table S9. Yasawa best-fit model: AUTHORITY, TEMPORAL, SPATIAL, I.D., and STORY

Fixed effects					
Factor	β (Maximum likelihood estimate)	95% CI	Standard error	*z* value	Wald *p* value
AUTHORITY	.198	-.127 –.523	.166	1.19	.233
TEMPORAL	.484	.161 –.807	.165	2.94	.0033
SPATIAL	.733	.404 – 1.06	.168	4.36	<.0001
Random effects					
Factor	Variance estimate	Standard Deviation			
I.D.	1.05	1.03			
STORY	.038	.195			

We first fit a baseline model with variables AUTHORITY, TEMPORAL, SPATIAL, then added I.D., which significantly improved the fit of the model (LR statistic = 166.5, $p < .0001$). Single-term deletions revealed two fixed effects, TEMPORAL, and SPATIAL, to be significant (TEMPORAL: LR statistic =8.55, $p = .003$; SPATIAL: LR statistic = 19.0, $p < .0001$), whereas AUTHORITY did not reach significance (LR statistic = 1.35, $p = .245$).

Adding STORY significantly improved the fit of the model (LR statistic =4.21, $p = .040$). Adding SEX did not improve the model fit (LR statistic = .824, $p = .364$), nor did adding EDUCATION (LR statistic = 2.07, $p = .150$) or AGE (LR statistic = .660, $p = .417$). Thus, the final best-fit model included the fixed factors AUTHORITY, TEMPORAL, and SPATIAL, and

the random factors I.D. and STORY. Table S10 summarizes the parameter estimates of the models described above.

Table S10. Comparison of models for Yasawa data

Factor	Baseline β values (sig.)	Model2 β values (sig.)	Model3 β values (sig.)	Model4 β values (sig.)
AUTHORITY	0.20 *ns* ($p < 0.24$)	0.20 *ns* ($p < 0.24$)	0.20 *ns* ($p < 0.24$)	0.20 *ns* ($p < 0.24$)
TEMPORAL	0.48 **	0.48 **	0.48 **	0.48 **
SPATIAL	0.73 ***	0.73 ***	0.73 ***	0.73 ***
Sex	NA	*ns* ($p < 0.37$)	NA	NA
Education	NA	NA	*ns* ($p < 0.16$)	NA
Age	NA	NA	NA	*ns* ($p < 0.42$)
Overall fit of the model (-2LL)	NA	*ns* ($p < 0.37$)	*ns* ($p < 0.16$)	*ns* ($p < 0.42$)

*** < 0.001, ** < 0.01, * < 0.05, *ns* non-significant, NA non-applicable

Figure S4 illustrates STORY effects via conditional modes with 95% confidence intervals based on the conditional variance:

Figure S4. Effects of STORY in the Yasawa Sample

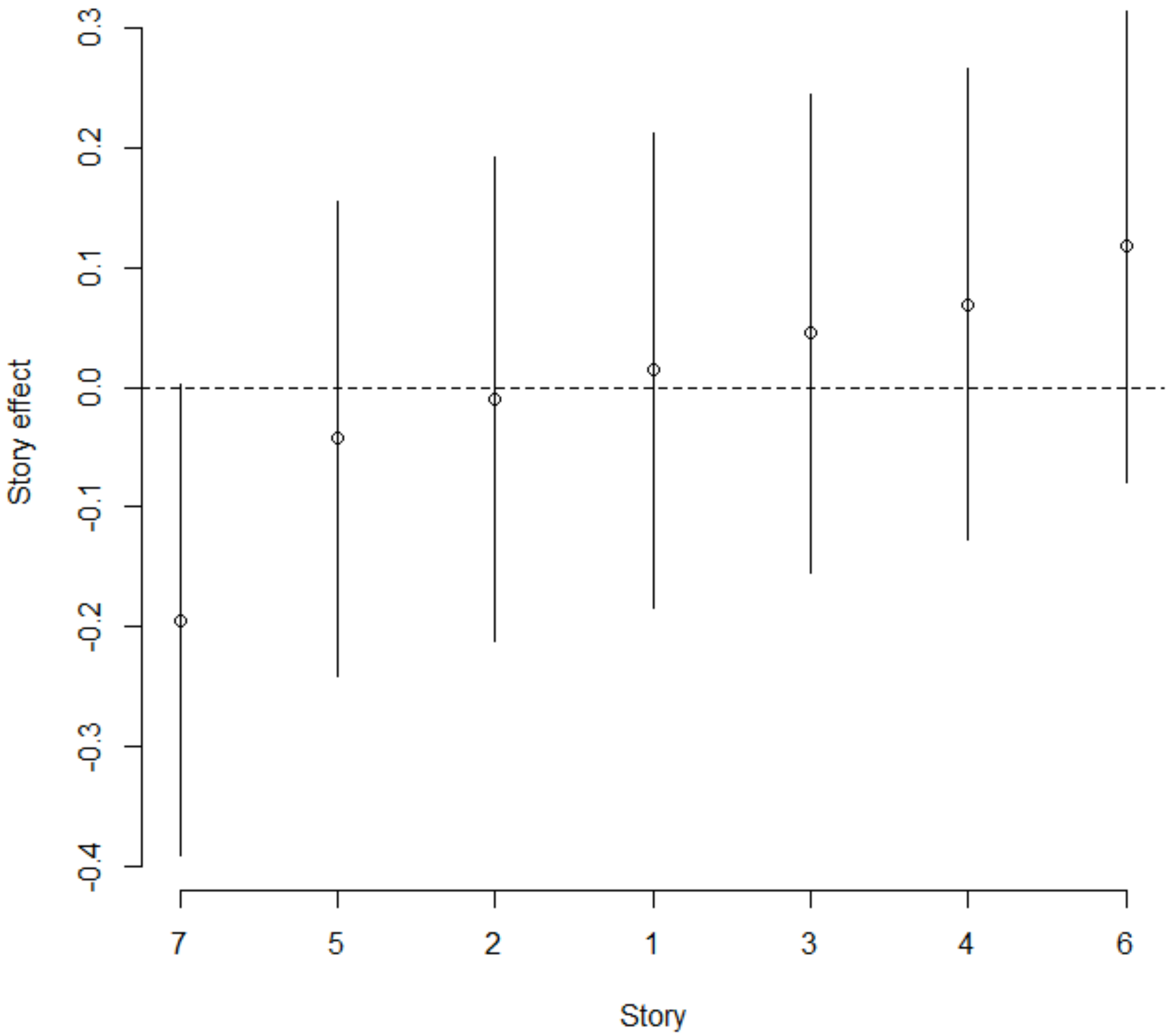

Yasawa participants' evaluations of Scenario 7 (Rape) are the least responsive to temporal or spatial distance or authority consent, while their evaluations of Scenario 6 (Unjust Perjury) show the greatest plasticity in this regard.

Karo Batak

The best-fit model for the Karo Batak included the fixed factors AUTHORITY, TEMPORAL, and SPATIAL, and the random factors I.D. and STORY. A summary of this model is shown in Table S11.

Table S11. Karo Batak best-fit model: AUTHORITY, TEMPORAL, SPATIAL, I.D., and STORY

Fixed effects					
Factor	β (Maximum likelihood estimate)	95% CI	Standard error	*z* value	Wald *p* value
AUTHORITY	1.42	.949 – 1.89	.241	5.90	<.0001
TEMPORAL	1.77	1.29 – 2.26	.248	7.14	<.0001
SPATIAL	2.27	1.76 – 2.78	.262	8.67	<.0001
Random effects					
Factor	Variance estimate	Standard Deviation			
I.D.	1.95	1.40			
STORY	.152	.389			

Again, we first fit a baseline model with variables AUTHORITY, TEMPORAL, SPATIAL, then added I.D., which significantly improved the fit of the model (LR statistic = 141.1, $p < .0001$). In addition, single-term deletions revealed all three fixed effects, AUTHORITY, TEMPORAL, and SPATIAL, to be significant (AUTHORITY: LR statistic =36.1, $p < .0001$; TEMPORAL: LR statistic = 56.6, $p < .0001$; SPATIAL: LR statistic = 84.5, $p < .0001$). Thus, there was significant variation in responses between individuals, as well as significant effects of each of our manipulated scenario variables.

We next added STORY, which significantly improved the fit of the model (LR statistic = 8.74, $p = .0031$). Adding SEX did not improve the model fit (LR statistic = .0099, $p = .921$), nor

did adding EDUCATION (LR statistic = .707, p = .401) or AGE (LR statistic = .042, p = .837). Thus, the final best-fit model included the fixed factors AUTHORITY, TEMPORAL, and SPATIAL, and the random factors I.D. and STORY. Table S12 summarizes the parameter estimates of the models described above.

Table S12. Comparison of models for Karo Batak data

Factor	Baseline β values (sig.)	Model2 β values (sig.)	Model3 β values (sig.)	Model4 β values (sig.)
AUTHORITY	1.42 ***	1.42 ***	1.42 ***	1.42 ***
TEMPORAL	1.77 ***	1.77 ***	1.77 ***	1.77 ***
SPATIAL	1.27 ***	1.27 ***	1.27 ***	1.27 ***
Sex	NA	*ns* (p < 0.92)	NA	NA
Education	NA	NA	*ns* (p < 0.41)	NA
Age	NA	NA	NA	*ns* (p < 0.84)
Overall fit of the model (-2LL)	NA	*ns* (p < 0.92)	*ns* (p < 0.41)	*ns* (p < 0.84)

*** < 0.001, ** < 0.01, * < 0.05, *ns* non-significant, NA non-applicable

Figure S5 illustrates STORY effects via conditional modes with 95% confidence intervals based on the conditional variance:

Figure S5. Effects of STORY in the Karo Batak Sample

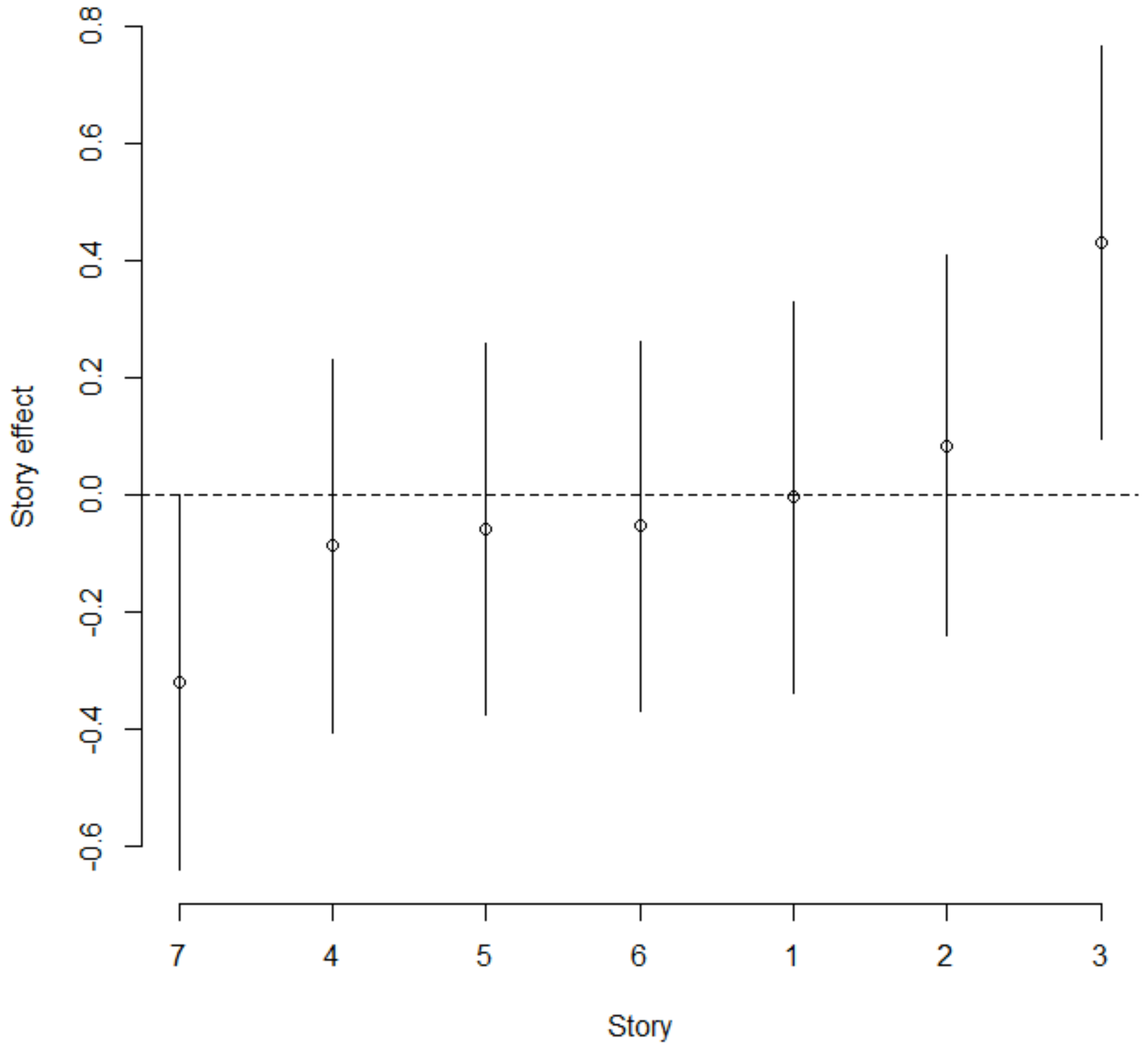

In the Karo Batak sample, evaluations of Scenario 7 (Rape) are least responsive to changes in temporal or spatial distance or authority consent, while Scenario 3, (Violence Following Accidental Harm), is highly responsive to such contextual factors. Participants' evaluations of the other scenarios display little or no effect in this regard.

Sursurunga

The best-fit model for the Sursurunga included the fixed factors AUTHORITY, TEMPORAL, and SPATIAL, and the random factors I.D. and STORY. A summary of this model is shown in Table S13.

Table S13. Sursurunga best-fit model: AUTHORITY, TEMPORAL, SPATIAL, I.D., and STORY

Fixed effects					
Factor	β (Maximum likelihood estimate)	95% CI	Standard error	*z* value	Wald *p* value
AUTHORITY	.386	-.088 –.861	.242	1.59	.111
TEMPORAL	.484	.010 –.957	.241	2.00	.0452
SPATIAL	.607	.135 – 1.08	.241	2.52	.0118
Random effects					
Factor	Variance estimate	Standard Deviation			
I.D.	3.01	1.74			
STORY	.481	.694			

We first fit a baseline model with variables AUTHORITY, TEMPORAL, SPATIAL, then added I.D., which significantly improved the fit of the model (LR statistic = 238.4, $p < .0001$). Single-term deletions revealed two fixed effects, TEMPORAL, and SPATIAL, to be significant (TEMPORAL: LR statistic =4.04, $p = .045$; SPATIAL: LR statistic = 6.41, $p = .011$), whereas AUTHORITY did not reach significance (LR statistic = 2.55, $p = .11$).

Adding STORY significantly improved the fit of the model (LR statistic =42.3, $p < .0001$). Adding SEX did not improve the model fit (LR statistic = .026, $p = .873$), nor did adding EDUCATION (LR statistic = 1.49, $p = .222$) or AGE (LR statistic = 2.03, $p = .154$). Thus, the final best-fit model included the fixed factors AUTHORITY, TEMPORAL, and SPATIAL, and

the random factors I.D. and STORY. Table S14 summarizes the parameter estimates of the models described above.

Table S14. Comparison of models for Sursurunga data

Factor	Baseline β values (sig.)	Model2 β values (sig.)	Model3 β values (sig.)	Model4 β values (sig.)
AUTHORITY	0.39 *ns* ($p < 0.12$)	0.39 *ns* ($p < 0.12$)	0.39 *ns* ($p < 0.12$)	0.39 *ns* ($p < 0.12$)
TEMPORAL	0.48 *	0.48 *	0.48 *	0.48 *
SPATIAL	0.61 *	0.61 *	0.61 *	0.61 *
Sex	NA	*ns* ($p < 0.88$)	NA	NA
Education	NA	NA	*ns* ($p < 0.23$)	NA
Age	NA	NA	NA	*ns* ($p < 0.16$)
Overall fit of the model (-2LL)	NA	*ns* ($p < 0.88$)	*ns* ($p < 0.23$)	*ns* ($p < 0.16$)

*** < 0.001, ** < 0.01, * < 0.05, *ns* non-significant, NA non-applicable

Figure S6 illustrates STORY effects via conditional modes with 95% confidence intervals based on the conditional variance:

Figure S6. Effects of STORY in the Sursurunga Sample

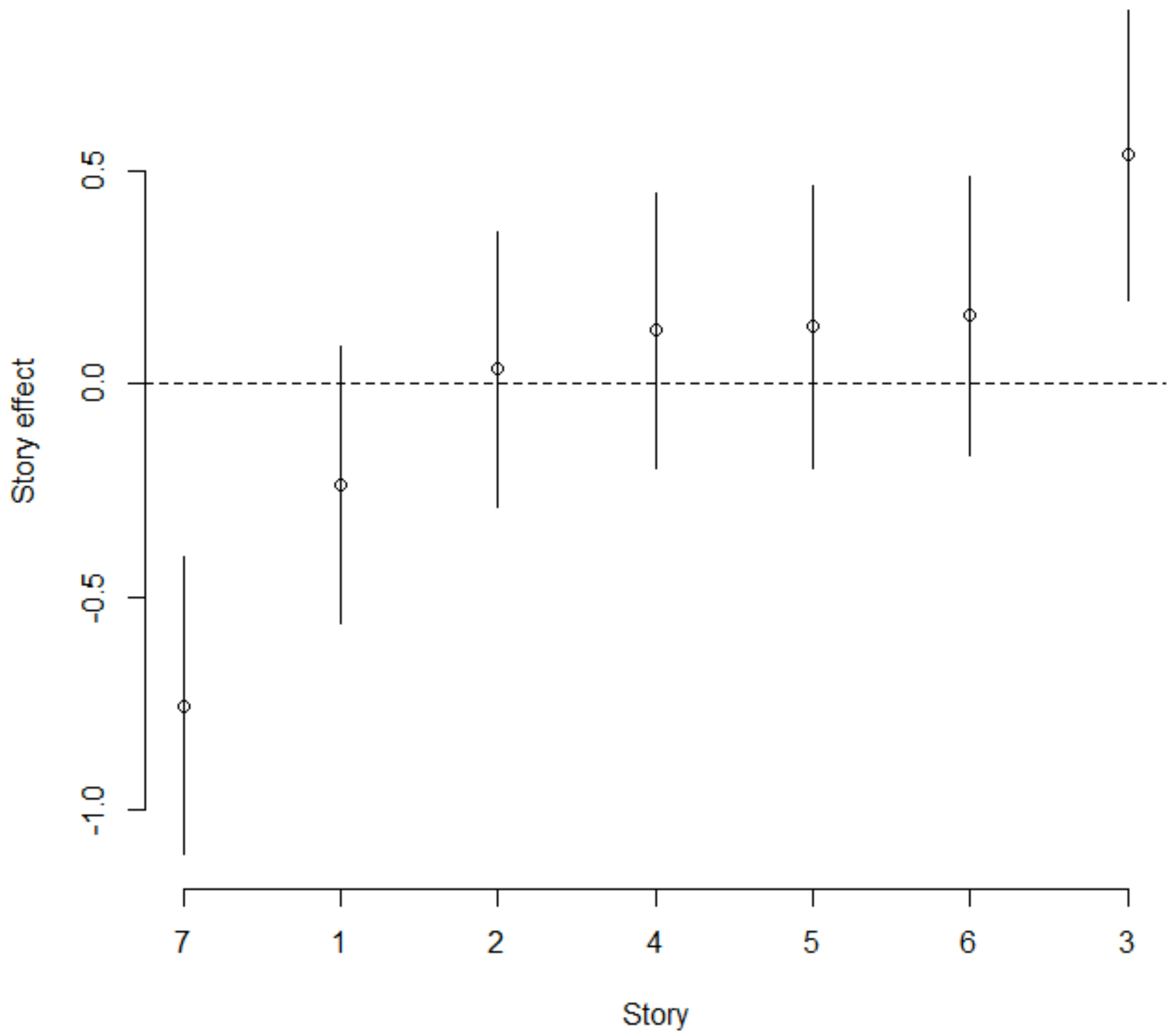

Sursurunga participants' evaluations of Scenario 7 (Rape) are the least responsive to changes in temporal distance, spatial distance, or authority consent, while their evaluations of Scenario 3 (Violence Following Accidental Harm) are the most responsive in this regard.

Storozhnitsa

The best-fit model for Storozhnitsa included the fixed factors AUTHORITY, TEMPORAL, and SPATIAL, and the random factors I.D. and STORY. A summary of this model is shown in Table S15.

Table S15. Storozhnitsa best-fit model: AUTHORITY, TEMPORAL, SPATIAL, I.D., and STORY

Fixed effects					
Factor	β (Maximum likelihood estimate)	95% CI	Standard error	*z* value	Wald *p* value
AUTHORITY	1.56	1.06 – 2.06	.255	6.13	<.0001
TEMPORAL	2.40	1.89 – 2.91	.261	9.21	<.0001
SPATIAL	2.39	1.88 – 2.90	.261	9.15	<.0001
Random effects					
Factor	Variance estimate	Standard Deviation			
I.D.	1.97	1.41			
STORY	.455	.675			

We first fit a baseline model with variables AUTHORITY, TEMPORAL, SPATIAL, then added I.D., which significantly improved the fit of the model (LR statistic = 150.7, $p < .0001$). In addition, single-term deletions revealed all three fixed effects, AUTHORITY, TEMPORAL, and SPATIAL, to be significant (AUTHORITY: LR statistic =38.0, $p < .0001$; TEMPORAL: LR statistic = 92.6, $p < .0001$; SPATIAL: LR statistic = 91.4, $p < .0001$). Thus, there was significant variation in responses between individuals, as well as significant effects of each of our manipulated scenario variables.

We next added STORY, which significantly improved the fit of the model (LR statistic =46.7, $p < .0001$). Adding SEX did not improve the model fit (LR statistic = 1.64, $p = .20$), nor

did adding EDUCATION (LR statistic = .110, $p = .74$) or AGE (LR statistic = .219, $p = .64$). Thus, the final best-fit model included the fixed factors AUTHORITY, TEMPORAL, and SPATIAL, and the random factors I.D. and STORY. Table S16 summarizes the parameter estimates of the models described above.

Table S16. Comparison of models for Storozhnitsa data

Factor	Baseline β values (sig.)	Model2 β values (sig.)	Model3 β values (sig.)	Model4 β values (sig.)
AUTHORITY	1.56 ***	1.56 ***	1.56 ***	1.56 ***
TEMPORAL	2.40 ***	2.40 ***	2.40 ***	2.40 ***
SPATIAL	2.39 ***	2.39 ***	2.39 ***	2.39 ***
Sex	NA	*ns* (p < 0.21)	NA	NA
Education	NA	NA	*ns* (p < 0.75)	NA
Age	NA	NA	NA	*ns* (p < 0.64)
Overall fit of the model (-2LL)	NA	*ns* (p < 0.21)	*ns* (p < 0.75)	*ns* (p < 0.64)

*** < 0.001, ** < 0.01, * < 0.05, *ns* non-significant, NA non-applicable

Figure S7 illustrates STORY effects via conditional modes with 95% confidence intervals based on the conditional variance:

Figure S7. Effects of STORY in the Storozhnitsa Sample

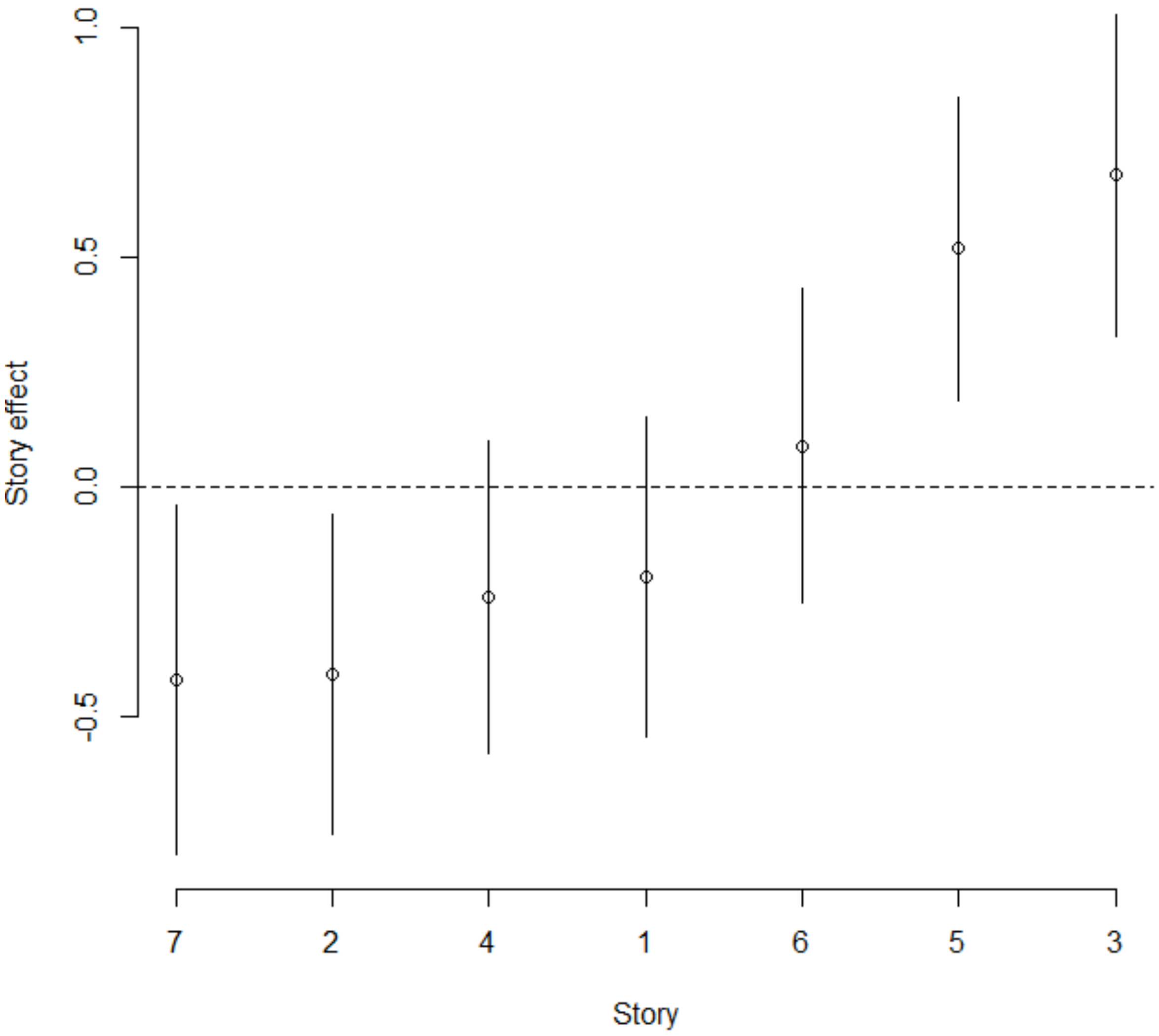

Storozhnitsa participants' evaluations of Scenarios 7 (Rape) and 2 (Wife Battery) are least responsive to changes in temporal or spatial distance or authority consent, while evaluations of Scenarios 5 (Defamation) and 3 (Violence Following Accidental Harm) are the most responsive in this regard.

Urban California

The best-fit model for California included the fixed factors AUTHORITY, TEMPORAL, and SPATIAL, and the random factors I.D. and STORY. A summary of this model is shown in Table S17.

Table S17. California best-fit model: AUTHORITY, TEMPORAL, SPATIAL, I.D., and STORY

Fixed effects					
Factor	β (Maximum likelihood estimate)	95% CI	Standard error	*z* value	Wald *p* value
AUTHORITY	1.22	.731 – 1.70	.247	4.92	<.0001
TEMPORAL	1.17	.694 – 1.65	.245	4.79	<.0001
SPATIAL	1.26	.777 – 1.74	.245	5.13	<.0001
Random effects					
Factor	Variance estimate	Standard Deviation			
I.D.	2.97	1.72			
STORY	2.48	1.57			

We first fit a baseline model with variables AUTHORITY, TEMPORAL, SPATIAL, then added I.D., which significantly improved the fit of the model (LR statistic = 166.1, $p < .0001$). In addition, single-term deletions revealed all three fixed effects, AUTHORITY, TEMPORAL, and SPATIAL, to be significant (AUTHORITY: LR statistic =17.2, $p < .0001$; TEMPORAL: LR statistic = 16.8, $p < .0001$; SPATIAL: LR statistic = 19.3, $p < .0001$). Thus, there was significant variation in responses between individuals, as well as significant effects of each of our manipulated scenario variables.

Adding STORY significantly improved the fit of the model (LR statistic =242.1, $p < .0001$). Adding SEX did not improve the model fit (LR statistic = .272, $p = .602$), nor did adding

EDUCATION (LR statistic = 1.95, p = .163) or AGE (LR statistic = .338, p = .561). Thus, the final best-fit model included the fixed factors AUTHORITY, TEMPORAL, and SPATIAL, and the random factors I.D. and STORY. Table S18 summarizes the parameter estimates of the models described above.

Table S18. Comparison of models for California data

Factor	Baseline β values (sig.)	Model2 β values (sig.)	Model3 β values (sig.)	Model4 β values (sig.)
AUTHORITY	1.22 ***	1.22 ***	1.22 ***	1.22 ***
TEMPORAL	1.17 ***	1.17 ***	1.17 ***	1.17 ***
SPATIAL	1.26 ***	1.26 ***	1.26 ***	1.26 ***
Sex	NA	*ns* (p < 0.61)	NA	NA
Education	NA	NA	*ns* (p < 0.17)	NA
Age	NA	NA	NA	*ns* (p < 0.57)
Overall fit of the model (-2LL)	NA	*ns* (p < 0.61)	*ns* (p < 0.17)	*ns* (p < 0.57)

*** < 0.001, ** < 0.01, * < 0.05, *ns* non-significant, NA non-applicable

Figure S8 illustrates STORY effects via conditional modes with 95% confidence intervals based on the conditional variance:

Figure S8. Effects of STORY in the California Sample

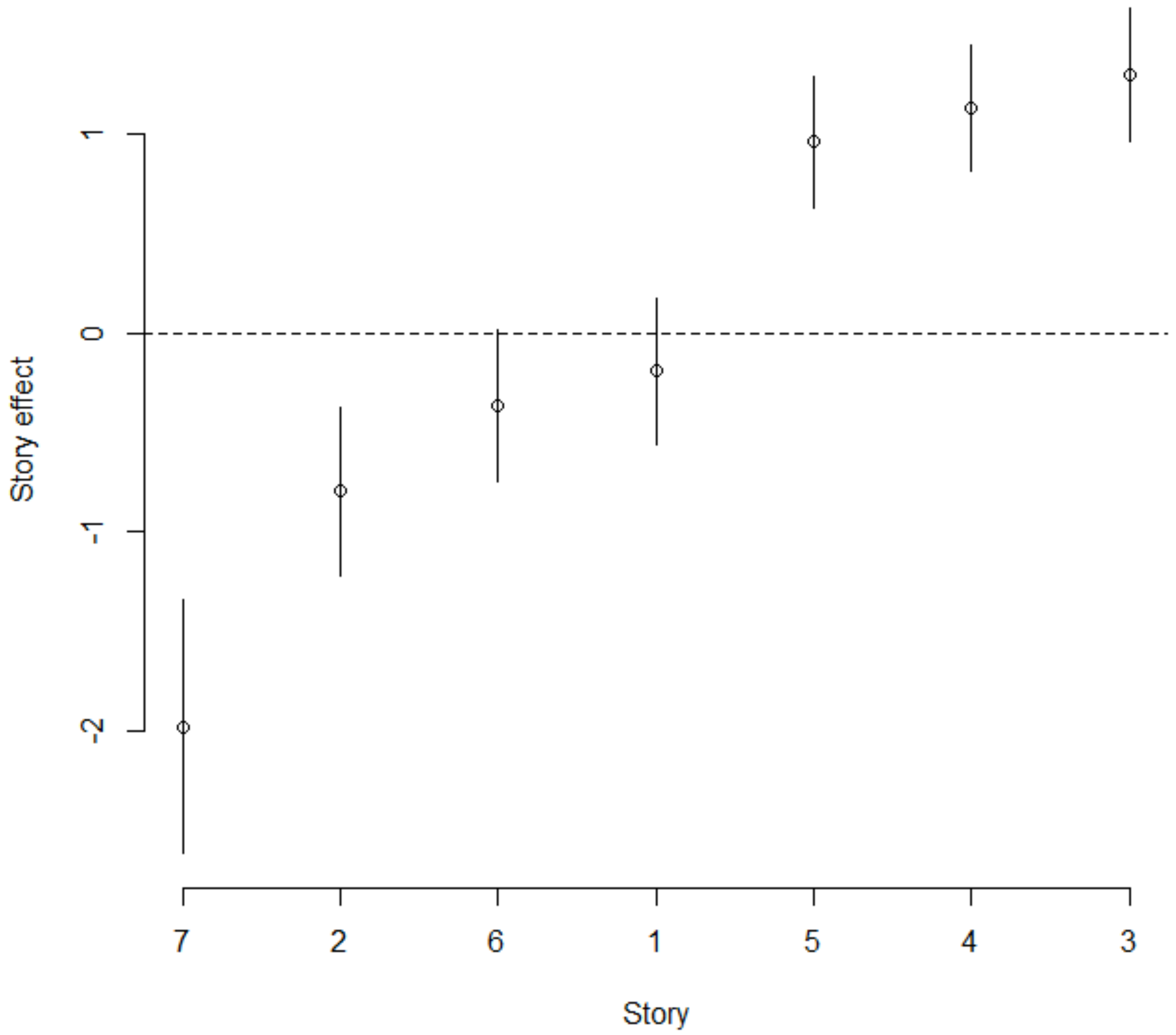

Californian participants' responses to Scenarios 7 (Rape), 2 (Wife Battery), and 6 (Unjust Perjury) displayed the least effect of changes in temporal or spatial distance or authority consent, while Scenarios 5 (Defamation), 4 (Marketplace Cheating), and 3 (Violence Following Accidental Harm) show the greatest plasticity in this regard.

Additional Considerations: Order of Presentation of Dependent Measures

To minimize the complexity of administering the protocol in the field, the key dependent measures were presented in fixed order: participants were first asked to judge the given act in the here and now, and were then asked to evaluate the act in light of manipulations of authority consent, temporal distance, and spatial distance. Might the reductions in assessed wrongness as a function of these manipulations reflect demand characteristics, such that, having indicated that the given act was unacceptable, participants interpreted the follow-up questions as prompting them to alter their responses? Were this the case, we would expect systematic changes in responses across the three questions. Although inspection of Fig. 1 reveals no consistent patterns in this regard, firm conclusions are not merited given that, by virtue of the use of a fixed order, order of presentation is confounded with the nature of the question. However, in a follow-up study, conducted in urban California and Seoul, Korea [6], employing nearly-identical materials and protocol (the Rape scenario was omitted, and a 9-point rating scale was used), but counterbalancing the order of presentation of the authority consent, temporal distance, and spatial distance questions, we both replicate the key results reported here and find no effect of question order, indicating that demand characteristics are unlikely to explain the present results.

Protocol Modifications by Field Site

As described below, a locally relevant authority was identified at each research site for use in the Authority Consent conditions. Scenario No.7, *Rape,* was not administered at the Shuar site, as this topic was deemed inappropriate given local sensibilities. Unless otherwise specified below, the researchers at each field site utilized the interview materials without further modification.

Tsimane’

The local authority was given as the *Gran Consejo Tsimane’,* which is a council of Tsimane’ men who represent Tsimane’ interests to outside political bodies, help arbitrate certain inter-community disputes, help organize pan-Tsimane’ meetings and projects, and adjudicate certain inter-village disputes. The *Gran Consejo* is the highest political rank obtainable in Tsimane’ society, but connotes limited prestige as many Tsimane’ see the *Gran Consejo* as supporting only certain villages and as subject to cronyism. The *Gran Consejo* has only been in existence since 1989, and has been fairly ineffective in stemming mistreatment of the Tsimane’ and exploitation of their resources. However, all Tsimane’ recognize the *Gran Consejo's* position. The authority of the *Gran Consejo* is limited, as governance is largely decentralized to villages, and even within villages extended families are highly autonomous

Shuar

The local authority was given as *los lideres de la comunidad,* "community leaders". This encompassing term was selected because, in Shuar communities, there is no person or persons who decides what is right or wrong, though people might be more inclined to listen if a plurality of respected people advance an opinion. The community leaders are the closest thing to a body that could make moral judgments, although most Shuar people would not view them as explicitly having that role. In Shuar villages, community leaders are elected in a town meeting, usually once every year or two. They are primarily drawn from the adult male *socios,* or adult male heads of household. There are also female socios, who are not typically elected village president, but who may serve in any of the other leadership roles of vice president, secretary, treasurer,

head of *padres de familia* (head of "village fathers") and head of *madres de familia* ("village mothers"). The head of the local school is usually considered a leader as well. The Shuar do not traditionally have chiefs or any official leadership outside heads of household. Hence, the village leadership might be most closely analogized to the leadership of a university department. They are there, for example, to facilitate official liaisons with government, convene meetings, or organize community projects. They do not have the power to do anything significant without village consensus, and have no power to punish. Community leaders have official legal power to take bureaucratic action on behalf of the village, but not policing power per se. However, they are able to call meetings in which the village decides a course of action, including punishing someone. Because the village president rotates, he is neither usually nor necessarily the highest status person in the village. Young men are often elected because they are viewed as having more youthful energy (and education, at times). Thus, village leadership could be regarded as having higher than median social status typically, but not any special official status beyond the respect they have garnered as individuals.

Karo Batak

The local authority was given as *Kepala Desa* ("Village Head"). The *Kepala Desa* is a legally elected official of relatively high prestige within village, as people depend on him for taking care of daily matters, such as official paperwork. The *Kepala Desa* settles disputes and deals with minor crimes, such as theft and non-lethal violence; the police are brought in to deal with serious crimes.

Sursurunga

The local authority employed at the Sursurunga site was *Pasta* ("Pastor"). The term is used by all Protestant denominations in the area, and refers to the local religious leader. Multiple smaller villages share a single *Pasta* between them, but most villages have their own, and some villages have *Pastas* from different denominations. *Pastas* rank fairly high within this society's relatively flat status hierarchy; the *Pasta's* input would be asked for in some contexts that are not directly religious – e.g., blessing a new Aid Post building. The *Pasta's* prestige varies considerably according to the degree to which the person conforms to local ideals of industriousness, intelligence, altruism, and affability. Nevertheless, a person in this position is assumed to be more or less a model citizen until evidence shows otherwise. Few people question the legitimacy of the *Pasta,* which is the result of a Grade 10 education (mean education in the area is around Grade 6) and theological training, as well as being selected by church authorities. The *Pasta* consistently makes pulpit claims about how people ought to live. These pronouncements are expected and hypocrisy is not well tolerated. Pulpit positions on moral issues cannot dictate behavior, but they can shape public opinion.

Yasawa

The local authority used in Yasawa was the *Turaga* ("Chief"). A chief holds the highest inherited rank within a very well-defined traditional village hierarchy. The Chief is the highest ranking person (usually the oldest male) in the highest ranked clan in a village. The office of the Chief is greatly respected, and is accorded public demonstrations of respect including spatial positioning, choice foods, serving order, honorific language, and elaborate funerary rites. The person holding the chiefly office is assumed to embody core Fijian ideals such as generosity, self-control, and respectfulness. However, individual Chiefs can lose the respect of the people, or

an heir can fail to be installed as a Chief, to the extent that he fails to embody these values. A Chief was traditionally viewed as possessing immense *mana*, a kind of force of efficacy that permeated his possessions and was dangerous to the touch. His traditional economic role entailed that he possess ample resources and distribute them generously based on need, and villagers in Yasawa still gather once a year to plant the Chief's yam garden, from which all can draw in times of need. Many Fijians currently lament the general loss of *mana* by all Fijians, and it does not appear to play much of a role in legitimating chiefly influence in modern-day Fiji. That said, the traditional lineage-based power structure of a village is still respected in the villages of Yasawa, and few question the right and responsibility of an exemplary Chief to influence village affairs. A village Chief is supposed to serve the good of the village, and it can fall to him to adjudicate disputes within the village when other options fail. However, a Chief cannot unilaterally change village rules without going through a village meeting, voicing a proposal to the villagers, and receiving support. For example, a prospective rule change might be introduced at a village meeting, the villagers would debate towards consensus, and the Chief would assent to the change and affirm its application. A Chief has little opportunity to dictate to villagers, and depends on prestige and consent for his influence.

Consonant with the above considerations, pilot testing revealed that, when presented with the original framing in the authority consent scenarios, Fijian participants inferred that (a) the Chief of the village made the act acceptable *after* it was committed, because (b) it was so severe that the Chief felt that he had to personally intervene to restore community order. Further, the Chief was (c) perceived as having unilaterally produced a novel normative claim, which the researchers were repeatedly told by the participants ran counter to the way a Fijian village is run. The Chief was therefore viewed as somewhat out of line – as noted above, the proper protocol

involves a village meeting, consensus building, and finally the Chief affirming the village consensus. In light of these discussions, in order to maximize ecological validity, the vignettes were modified so as to eliminate chronological ambiguity and conform to the village rule-making process. The Yasawa Authority Consent scenarios therefore read as follows:

> "Suppose that one year before [the transgression], [Actor's] village held a meeting to discuss village rules. At this meeting, everyone agreed that it is not bad [to commit this type of transgression], and so the Chief of the village affirmed this. If the Chief had affirmed that it is not bad to [to commit this type of transgression] one year before [Actor committed the transgression], then how good or bad would it be to do what [Actor] did?"

<u>Storozhnitsa</u>

The local authority used in Storozhnitsa was *Golova Sela* ("the Head of the Village"). The *Golova Sela* is the legally elected mayor. The title connotes relatively high prestige because he is elected and people depend on his help in dealing with many issues concerning their daily lives (such as various official permissions, etc.). The *Golova Sela* does not settle violent acts, stealing or other crimes, but does adjudicate small property disputes. In general, the *Golova Sela* is not called on to settle breaches of laws / rules / norms / conventions. However, the *Golova Sela's* advice may be sought regarding negotiations with other state authorities, including the Police and Justice officials, after such transgressions.

<u>Urban California</u>

In the urban California sample, the local authority was designated as unspecified "community leaders," as pilot testing revealed that participants balked at the implausibility of, for example, the Mayor, City Council, or Police Chief legalizing battery or theft. In addition, the phrase "while camping near the town" was added to explain the rural backdrop of the Stealing Scenario, and the victim's country of origin was changed from Iceland to Tuva, as, from the American perspective, Iceland is a relatively culturally similar Western nation. In the Market Cheating scenario, the foreign stranger was said to come from Laos. In the Rape scenario, the city to which the rapist travels was Seattle, and the victim was described as "not an American".

Complete Protocol

Participants at each site were presented with the transgression scenarios in one of two orders, balanced between participants (for convenience, elsewhere in the ESM, scenarios are identified using numbers derived from Order 1).

Order 1	Order 2
1. Stealing	1. Unjust Perjury
2. Wife Battery	2. Defamation
3. Violence Following Accidental Harm	3. Marketplace Cheating
4. Marketplace Cheating	4. Violence Following Accidental Harm
5. Defamation	5. Wife Battery
6. Unjust Perjury	6. Stealing
7. Rape	7. Rape

Preamble to Participants

I would like to read you some stories about events that occurred in a [insert ethnic/language community name, e.g., Shuar] community like this one, and then ask you some questions about each story. It will take about 35 minutes, and you will receive [INSERT LOCAL PAYMENT] as soon as we are finished. You do not have to participate, or we can schedule for later if you do not have time now. If we start and then you decide that you would prefer not to participate, that is fine, and we can stop whenever you want. Are you willing to participate?

1. STEALING

A [locally appropriate male name] is a man from this other [insert ethnic/language community name, e.g. Shuar] community. On a road near the village, A encounters a stranger from Iceland, a country that is very far away from here [if Iceland is problematic in a given field site, investigators may replace it with some other country or known place that is far away]. The stranger does not speak [language spoken by the subject]. After the stranger passes A, the stranger puts his sack down and walks down a small hill to wash in a stream. When the stranger is out of sight, A opens his sack and looks at the contents. He finds [roughly a week's wages locally], takes the money and walks away quickly. The stranger does not realize his money has been taken until he is back home in his country, and he is then too far away to do anything about it.

STEALING: Comprehension Questions

"Because it is important that I make myself clear, I'd like to make sure that you understood me. Could you please tell me what happened in the story I just told you?"
[RA: Listen to the participant's response, and check off any of the three concepts that are present in the participant's initial summary of the story by placing a 1 in the column. For each item that is not present in the initial summary, place a 0 in the column and ask the corresponding probe question, then record in the column whether the participant answered the question correctly; use a 1 for correct and a 0 for incorrect.]

Concept	Mentioned in P's 1st summary?	Question if Not mentioned	Correct?	Correct?
Stole from stranger		From whom did A steal?		
Stranger different group		Was the stranger a member of the [local ethnic/language group]?		
Time delay in detection		Did the stranger detect that his money had been stolen while he was in [Insert local country]?		

[RA: If the participant gets one or more of the probes wrong, say "Perhaps I did not make myself clear. Please let me read the story to you again;" then return to the story and re-read it once more. Then, go through the probes that the subject got wrong again, recording the responses in the column (1 for a correct answer, 0 for an incorrect answer). After round 2, regardless of whether they still make mistakes, proceed to questions below.]

STEALING: Questions

[Severity]	How good or bad is what A did? Please show me on this line. [Show subjects standard severity judgment scale.]<br>[Response:] __________ [record number from scale; If the participant indicates an intermediate point on the scale, record the nearest whole number value.]

[Justification] Why? [Record notes on participant's response here:]

[Reputation]	What would people think of A? Would they think he is a good person or a bad person? Please

	show me on this line. [Show subjects standard severity judgment scale.] [Response:] _________ [record number from scale; If the participant indicates an intermediate point on the scale, record the nearest whole number value.]

[Authority Dependence]	[Replace X with the name of a local authority figure type or group that is the most natural authority for moral issues of this kind. This authority type might be "Head of the Village", or "Council of Elders," or "Pastor". Note: This should not be the personal name of an individual – it should be the name of a type of authority]<br><br>Suppose that X said that it is not bad to take things from strangers who do not live nearby and do not speak [insert name of the language spoken by the subject]. If X said that, how good or bad would it be to do what A did? Please show me on this line. [Show subjects standard severity judgment scale.]<br>[Response:] _________ [record number from scale; If the participant indicates an intermediate point on the scale, record the nearest whole number value.]

[Long Ago]	What if this happened a long, long time ago, before your grandparents were born, even before their grandparents were born. How good or bad would it be to do what A did a very long time ago? Please show me on this line. [Show subjects standard severity judgment scale.]<br>[Response:] _________ [record number from scale; If the participant indicates an intermediate point on the scale, record the nearest whole number value.]

[Far Away]	What if this happened in place very far from here, a place that no one in this village has ever visited, and I (the experimenter) have never visited either. How good or bad would it be to do what A did if it happened very far from here? Please show me on this line. [Show subjects standard severity judgment scale.]<br>[Response:] _________ [record number from scale; If the participant indicates an intermediate point on the scale, record the nearest whole number value.]

2. WIFE BATTERY

In this other [insert ethnic/language community name, e.g. Shuar] community, A [use an appropriate male name] returns home feeling very angry because his belongings have been damaged in a storm. His wife, who he knows was not responsible for the damage to his belongings, greets him warmly as he enters the house. Because he is angry, he slaps her face very hard, causing her nose to bleed.

WIFE BATTERY: Comprehension Questions

[RA: Read the following to the subject]
"Because it is important that I make myself clear, I'd like to make sure that you understood me. Could you please tell me what happened in the story I just told you?"

[RA: Listen to the participant's response, and check off any of the three concepts that are present in the participant's initial summary of the story by placing a 1 in the column. For each item that is not present in the initial summary, place a 0 in the column and ask the corresponding probe question, then record in the column whether the participant answered the question correctly; use a 1 for correct and a 0 for incorrect.]

**Concept**	**Mentioned in P's 1st Summary?**	**Question if Not mentioned**	**Correct?**	**Correct?**
Belongings damaged		What happened to A's belongings?		

Wife not responsible		Was A's wife responsible for the damage to A's belongings?		
Slapped his wife		What did A do to his wife?		

[RA: If the participant gets one or more of the probes wrong, say "Perhaps I did not make myself clear. Please let me read the story to you again;" then return to the story and re-read it once more. Then, go through the probes that the subject got wrong again, recording the responses in the column (1 for a correct answer, 0 for an incorrect answer). After round 2, regardless of whether they still make mistakes, proceed to questions below.]

WIFE BATTERY: Questions

[Severity]	How good or bad is what A did? Please show me on this line. [Show subjects standard severity judgment scale.]<br>[Response:] _________ [record number from scale; If the participant indicates an intermediate point on the scale, record the nearest whole number value.]

[Justification] Why? [Record notes on participant's response here:]

[Reputation]	What would people think of A? Would they think he is a good person or a bad person? Please show me on this line. [Show subjects standard severity judgment scale.]<br>[Response:] _________ [record number from scale; If the participant indicates an intermediate point on the scale, record the nearest whole number value.]

[Authority Dependence]	[Replace X with the name of a local authority figure type or group that is the most natural authority for moral issues of this kind. This authority type might be "Head of the Village", or "Council of Elders," or "Pastor". Note: This should not be the personal name of an individual – it should be the name of a type of authority]<br><br>Suppose that X said that it is not bad for a man to slap his wife if he is angry. If X said that, how good or bad would it be to do what A did? Please show me on this line.<br>[Show subjects standard severity judgment scale.]<br>[Response:] _________ [record number from scale; If the participant indicates an intermediate point on the scale, record the nearest whole number value.]

[Long Ago]	What if this happened a long, long time ago, before your grandparents were born, even before their grandparents were born. How good or bad would it be to do what A did a very long time ago? Please show me on this line. [Show subjects standard severity judgment scale.]<br>[Response:] _________ [record number from scale; If the participant indicates an intermediate point on the scale, record the nearest whole number value.]

[Far Away]	What if this happened in place very far from here, a place that no one in this village has ever visited, and I (the experimenter) have never visited either. How good or bad would it be to do what A did if it happened very far from here? Please show me on this line. [Show subjects standard severity judgment scale.]<br>[Response:] _________ [record number from scale; If the participant indicates an intermediate point on the scale, record the nearest whole number value.]

3. VIOLENCE FOLLOWING ACCIDENTAL HARM

In this other [insert ethnic/language community name, e.g. Shuar] community, A and B [use appropriate male names] are walking on a very muddy path. A slips in the mud and accidentally knocks B down as he tries to regain

his balance. B injures his arm in the fall. B knows that it was an accident. When he gets up B is very angry and hits A in the face.

VIOLENCE FOLLOWING ACCIDENTAL HARM: Comprehension Questions

[RA: Read the following to the subject]
"Because it is important that I make myself clear, I'd like to make sure that you understood me. Could you please tell me what happened in the story I just told you?"

[RA: Listen to the participant's response, and check off any of the three concepts that are present in the participant's initial summary of the story by placing a 1 in the column. For each item that is not present in the initial summary, place a 0 in the column and ask the corresponding probe question, then record in the column whether the participant answered the question correctly; use a 1 for correct and a 0 for incorrect.]

**Concept**	**Mentioned in P's 1st Summary?**	**Question if Not mentioned**	**Correct?**	**Correct?**
Intention		Did A knock B down on purpose?		
Injury		Was B injured in the fall?		
Physical Retribution		What did B do to A after he got back up?		

[RA: If the participant gets one or more of the probes wrong, say "Perhaps I did not make myself clear. Please let me read the story to you again;" then return to the story and re-read it once more. Then, go through the probes that the subject got wrong again, recording the responses in the column (1 for a correct answer, 0 for an incorrect answer). After round 2, regardless of whether they still make mistakes, proceed to questions below.]

VIOLENCE FOLLOWING ACCIDENTAL HARM: Questions

[Severity]	How good or bad is what B did? Please show me on this line. [Show subjects standard severity judgment scale.]<br>[Response:] __________ [record number from scale; If the participant indicates an intermediate point on the scale, record the nearest whole number value.]

[Justification]   Why? [Record notes on participant's response here:]

[Reputation]	What would people think of B? Would they think he is a good person or a bad person? Please show me on this line. [Show subjects standard severity judgment scale.]<br>[Response:] __________ [record number from scale; If the participant indicates an intermediate point on the scale, record the nearest whole number value.]

[Authority Dependence]	[Replace X with the name of a local authority figure type or group that is the most natural authority for moral issues of this kind. This authority type might be "Head of the Village", or "Council of Elders," or "Pastor". Note: This should not be the personal name of an individual – it should be the name of a type of authority]<br><br>Suppose that X said that it is not bad for a man to hit another person if that person causes an injury, even when the injury was not caused intentionally. If X said that, how good or bad would

	it be to do what B did? Please show me on this line.<br>[Show subjects standard severity judgment scale.]<br>[Response:] _________ [record number from scale; If the participant indicates an intermediate point on the scale, record the nearest whole number value.]

[Long Ago]	What if this happened a long, long time ago, before your grandparents were born, even before their grandparents were born. How good or bad would it be to do what B did a very long time ago? Please show me on this line. [Show subjects standard severity judgment scale.]<br>[Response:] _________ [record number from scale; If the participant indicates an intermediate point on the scale, record the nearest whole number value.]

[Far Away]	What if this happened in place very far from here, a place that no one in this village has ever visited, and I (the experimenter) have never visited either. How good or bad would it be to do what B did if it happened very far from here? Please show me on this line. [Show subjects standard severity judgment scale.]<br>[Response:] _________ [record number from scale; If the participant indicates an intermediate point on the scale, record the nearest whole number value.]

4. MARKETPLACE CHEATING

In this other [insert ethnic/language community name, e.g. Shuar] community, one day a stranger comes to buy food from A [in the market, where appropriate]. The stranger is not a local person and does not speak [insert the name of the language spoken by the subject]. The stranger comes from Iceland, a country that is very far from here. [If Iceland is problematic in a given field site, investigators may replace it with some other country or known place that is far away and unproblematic.] The stranger buys some fresh food from A and pays A the amount agreed. However, A does not give the stranger the fresh food that the stranger had chosen. Instead, he replaces the fresh food that the stranger had chosen with older food that is beginning to have a rotten smell. He wraps the food in a package so that the stranger is not aware of the switch until he is far away.

MARKETPLACE CHEATING: Comprehension Questions

[RA: Read the following to the subject]
"Because it is important that I make myself clear, I'd like to make sure that you understood me. Could you please tell me what happened in the story I just told you?"
[RA: Listen to the participant's response, and check off any of the three concepts that are present in the participant's initial summary of the story by placing a 1 in the column. For each item that is not present in the initial summary, place a 0 in the column and ask the corresponding probe question, then record in the column whether the participant answered the question correctly; use a 1 for correct and a 0 for incorrect.]

**Concept**	**Mentioned in P's 1st Summary?**	**Question if Not mentioned**	**Correct?**	**Correct?**
Non Local		Was the stranger [insert local ethnic group]?		
Received purchased goods		Did the stranger get the goods that he paid for?		
Time delay		When did the stranger become aware of the switch?		

[RA: If the participant gets one or more of the probes wrong, say "Perhaps I did not make myself clear. Please let me read the story to you again;" then return to the story and re-read it once more. Then, go through the probes that the subject got wrong again, recording the responses in the column (1 for a correct answer, 0 for an incorrect answer). After round 2, regardless of whether they still make mistakes, proceed to questions below.]

MARKETPLACE CHEATING: Questions

[Severity]	How good or bad is what A did? Please show me on this line. [Show subjects standard severity judgment scale.]<br>[Response:] _________ [record number from scale; If the participant indicates an intermediate point on the scale, record the nearest whole number value.]

[Justification] Why? [Record notes on participant's response here:]

[Reputation]	What would people think of A? Would they think he is a good person or a bad person? Please show me on this line. [Show subjects standard severity judgment scale.]<br>[Response:] _________ [record number from scale; If the participant indicates an intermediate point on the scale, record the nearest whole number value.]

[Authority Dependence]	[Replace X with the name of a local authority figure type or group that is the most natural authority for moral issues of this kind. This authority type might be "Head of the Village", or "Council of Elders," or "Pastor". Note: This should not be the personal name of an individual – it should be the name of a type of authority]<br><br>Suppose that X said that it is not bad for a man cheat another man in the market if the other man is not local and does not speak L [the language spoken by the subject]. If X said that, how good or bad would it be to do what A did? Please show me on this line.<br>[Show subjects standard severity judgment scale.]<br>[Response:] _________ [record number from scale; If the participant indicates an intermediate point on the scale, record the nearest whole number value.]

[Long Ago]	What if this happened a long, long time ago, before your grandparents were born, even before their grandparents were born. How good or bad would it be to do what A did a very long time ago? Please show me on this line. [Show subjects standard severity judgment scale.]<br>[Response:] _________ [record number from scale; If the participant indicates an intermediate point on the scale, record the nearest whole number value.]

[Far Away]	What if this happened in place very far from here, a place that no one in this village has ever visited, and I (the experimenter) have never visited either. How good or bad would it be to do what A did if it happened very far from here? Please show me on this line. [Show subjects standard severity judgment scale.]<br>[Response:] _________ [record number from scale; If the participant indicates an intermediate point on the scale, record the nearest whole number value.]

5. DEFAMATION

In this other [insert ethnic/language community name, e.g. Shuar] community, A and B [use appropriate male names] are neighbors. However, they do not like each other. One day, A decides to spread a false rumor that B has been stealing from others in the community. Though the rumor is not true, many people in the village believe it and it does great damage to B's reputation.

DEFAMATION: Comprehension Questions

[RA: Read the following to the subject]
"Because it is important that I make myself clear, I'd like to make sure that you understood me. Could you please tell me what happened in the story I just told you?"

[RA: Listen to the participant's response, and check off any of the three concepts that are present in the participant's initial summary of the story by placing a 1 in the column. For each item that is not present in the initial summary, place a 0 in the column and ask the corresponding probe question, then record in the column whether the participant answered the question correctly; use a 1 for correct and a 0 for incorrect.]

**Concept**	**Mentioned in P's 1st Summary?**	**Question if Not mentioned**	**Correct?**	**Correct?**
Disliked each other		Did A and B like each other, or dislike each other?		
False Rumor		Was the rumor about B true?		
Damage to Reputation		What happened to B's reputation?		

[RA: If the participant gets one or more of the probes wrong, say "Perhaps I did not make myself clear. Please let me read the story to you again;" then return to the story and re-read it once more. Then, go through the probes that the subject got wrong again, recording the responses in the column (1 for a correct answer, 0 for an incorrect answer). After round 2, regardless of whether they still make mistakes, proceed to questions below.]

DEFAMATION: Questions

[Severity]	How good or bad is what A did? Please show me on this line. [Show subjects standard severity judgment scale.]<br>[Response:] __________ [record number from scale; If the participant indicates an intermediate point on the scale, record the nearest whole number value.]

[Justification] Why? [Record notes on participant's response here:]

[Reputation]	What would people think of A? Would they think he is a good person or a bad person? Please show me on this line. [Show subjects standard severity judgment scale.]<br>[Response:] __________ [record number from scale; If the participant indicates an intermediate point on the scale, record the nearest whole number value.]

[Authority Dependence]	[Replace X with the name of a local authority figure type or group that is the most natural authority for moral issues of this kind. This authority type might be "Head of the Village", or "Council of Elders" or "Pastor". Note: This should not be the personal name of an individual – it should be the name of a type of authority]<br><br>Suppose that X said that it is not bad for a man to spread a false rumor about someone he does not like. If X said that, how good or bad would it be to do what A did? Please show me on this line.<br>[Show subjects standard severity judgment scale.]<br>[Response:] __________ [record number from scale; If the participant indicates an intermediate point on the scale, record the nearest whole number value.]

[Long Ago]	What if this happened a long, long time ago, before your grandparents were born, even before their grandparents were born. How good or bad would it be to do what A did a very long time ago? Please show me on this line. [Show subjects standard severity judgment scale.]<br>[Response:] _________ [record number from scale; If the participant indicates an intermediate point on the scale, record the nearest whole number value.]

[Far Away]	What if this happened in place very far from here, a place that no one in this village has ever visited, and I (the experimenter) have never visited either. How good or bad would it be to do what A did if it happened very far from here? Please show me on this line. [Show subjects standard severity judgment scale.]<br>[Response:] _________ [record number from scale; If the participant indicates an intermediate point on the scale, record the nearest whole number value.]

6. UNJUST PERJURY

A and B [use appropriate male names] are young men from this other [insert ethnic/language community name, e.g. Shuar] community. One night they have an argument and A starts a fight with B and seriously injures him. A few days later, there is a community meeting to discuss what should be done about the incident. Before the meeting, C, an influential man in the community, accepts money from A as a bribe [insert local term for bribe]. As a result, C lies in the meeting and says that B started the fight and everyone believes him. And so the community decides to punish B but not to punish A.

UNJUST PERJURY: Comprehension Questions

[RA: Read the following to the subject]
"Because it is important that I make myself clear, I'd like to make sure that you understood me. Could you please tell me what happened in the story I just told you?"

[RA: Listen to the participant's response, and check off any of the three concepts that are present in the participant's initial summary of the story by placing a 1 in the column. For each item that is not present in the initial summary, place a 0 in the column and ask the corresponding probe question, then record in the column whether the participant answered the question correctly; use a 1 for correct and a 0 for incorrect.]

**Concept**	**Mentioned in P's 1st Summary?**	**Question if Not mentioned**	**Correct?**	**Correct?**
A starts fight		Who started the fight?		
C lies		Did C tell the truth about who started the fight?		
A bribed C		Why did C lie for A?		

[RA: If the participant gets one or more of the probes wrong, say "Perhaps I did not make myself clear. Please let me read the story to you again;" then return to the story and re-read it once more. Then, go through the probes that the subject got wrong again, recording the responses in the column (1 for a correct answer, 0 for an incorrect answer). After round 2, regardless of whether they still make mistakes, proceed to questions below.]

UNJUST PERJURY: Questions

[Severity]	How good or bad is what C did? Please show me on this line. [Show subjects standard severity judgment scale.]<br>[Response:] _________ [record number from scale; If the participant indicates an intermediate

	point on the scale, record the nearest whole number value.]

[Justification] Why? [Record notes on participant's response here:]

[Reputation]	What would people think of C if they knew what he had done? Would they think he is a good person or a bad person? Please show me on this line. [Show subjects standard severity judgment scale.]<br>[Response:] _________ [record number from scale; If the participant indicates an intermediate point on the scale, record the nearest whole number value.]

[Authority Dependence]	[Replace X with the name of a local authority figure type or group that is the most natural authority for moral issues of this kind. This authority type might be "Head of the Village", or "Council of Elders," or "Pastor". Note: This should not be the personal name of an individual – it should be the name of a type of authority]<br><br>Suppose that X said that it is not bad for an influential person to speak in defense of an accused person in exchange for a bribe. If X said that, how good or bad would it be to do what C did? Please show me on this line.<br>[Show subjects standard severity judgment scale.]<br>[Response:] _________ [record number from scale; If the participant indicates an intermediate point on the scale, record the nearest whole number value.]

[Long Ago]	What if this happened a long, long time ago, before your grandparents were born, even before their grandparents were born. How good or bad would it be to do what C did a very long time ago? Please show me on this line. [Show subjects standard severity judgment scale.]<br>[Response:] _________ [record number from scale; If the participant indicates an intermediate point on the scale, record the nearest whole number value.]

[Far Away]	What if this happened in place very far from here, a place that no one in this village has ever visited, and I (the experimenter) have never visited either. How good or bad would it be to do what C did if it happened very far from here? Please show me on this line. [Show subjects standard severity judgment scale.]<br>[Response:] _________ [record number from scale; If the participant indicates an intermediate point on the scale, record the nearest whole number value.]

7. RAPE

A [use an appropriate male name] is a young man from this other [insert ethnic/language community name, e.g. Tsimane'] community, who goes to P [replace P with name of a city or large town in the region]. While A is in P, he meets B [use an appropriate female name], a woman from P who is not a [insert name of A's ethnic group, e.g., Tsimane']. A asks B for directions and she provides them to him. A wants to have sex with B, but she doesn't want to. A then forces B to have sex with him.

RAPE: Comprehension Questions

[RA: Read the following to the subject]
"Because it is important that I make myself clear, I'd like to make sure that you understood me. Could you please tell me what happened in the story I just told you?"

[RA: Listen to the participant's response, and check off any of the three concepts that are present in the participant's initial summary of the story by placing a 1 in the column. For each item that is not present in the initial summary,

place a 0 in the column and ask the corresponding probe question, then record in the column whether the participant answered the question correctly; use a 1 for correct and a 0 for incorrect.]

Concept	Mentioned in P's 1st Summary?	Question if Not mentioned	Correct?	Correct?
Gave directions		Why did B talk to A?		
B consent?		Did B want to have sex with A?		
Rape		What did A do to B?		

[RA: If the participant gets one or more of the probes wrong, say "Perhaps I did not make myself clear. Please let me read the story to you again;" then return to the story and re-read it once more. Then, go through the probes that the subject got wrong again, recording the responses in the column (1 for a correct answer, 0 for an incorrect answer). After round 2, regardless of whether they still make mistakes, proceed to questions below.]

RAPE: Questions

[Severity]	How good or bad is what A did? Please show me on this line. [Show subjects standard severity judgment scale.]<br>[Response:] _________ [record number from scale; If the participant indicates an intermediate point on the scale, record the nearest whole number value.]

[Justification] Why? [Record notes on participant's response here:]

[Reputation]	What would people think of A? Would they think he is a good person or a bad person? Please show me on this line. [Show subjects standard severity judgment scale.]<br>[Response:] _________ [record number from scale; If the participant indicates an intermediate point on the scale, record the nearest whole number value.]

[Authority Dependence]	[Replace X with the name of a local authority figure type or group that is the most natural authority for moral issues of this kind. This authority type might be "Head of the Village", or "Council of Elders," or "Pastor". Note: This should not be the personal name of an individual – it should be the name of a type of authority]<br><br>Suppose that X said that it is not bad for a man to force a woman to have sex if that woman is not a [insert name of A's ethnic group, e.g., Tsimane']. If X said that, how good or bad would it be to do what A did? Please show me on this line.<br>[Show subjects standard severity judgment scale.]<br>[Response:] _________ [record number from scale; If the participant indicates an intermediate point on the scale, record the nearest whole number value.]

[Long Ago]	What if this happened a long, long time ago, before your grandparents were born, even before their grandparents were born. How good or bad would it be to do what A did a very long time ago? Please show me on this line. [Show subjects standard severity judgment scale.]<br>[Response:] _________ [record number from scale; If the participant indicates an intermediate point on the scale, record the nearest whole number value.]

[Far Away]	What if this happened in place very far from here, a place that no one in this village has ever visited, and I (the experimenter) have never visited either. How good or bad would it be to do what A did if it happened very far from here? Please show me on this line. [Show subjects standard severity judgment scale.]<br>[Response:] _________ [record number from scale; If the participant indicates an intermediate point on the scale, record the nearest whole number value.]

Fig. S9. Scale used to indicate responses to key dependent measures

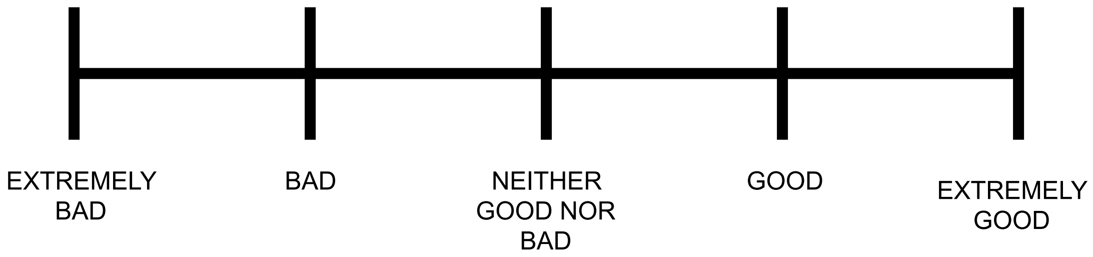

References

1. Henrich, J. & N. Henrich. 2010 The evolution of cultural adaptations: Fijian food taboos protect against dangerous marine toxins. *Proceedings of the Royal Society B: Biological Sciences* **277**: 3715-3724. (doi: 10.1098/rspb.2010.1191)
2. Bolyanatz, A. H. 2000 *Mortuary feasting on New Ireland: The activation of matriliny among the Sursurunga*. Greenwood Publishing Group.
3. Christensen, R. H. B. 2012 *A Tutorial on fitting Cumulative Link Models with the ordinal Package*. ftp://ftp.stat.math.ethz.ch/R-CRAN/web/packages/ordinal/vignettes/clm_tutorial.pdf.
4. R Core Team 2013 *A language and environment for statistical computing*Vienna: R Foundation for Statistical Computing. http://www.R-project.org.
5. Sousa, P. 2009 On testing the 'moral law'. *Mind & Language* **24**: 209-234. (DOI: 10.1111/j.1468-0017.2008.01360.x)
6. Holbrook, C., D. M. T. Fessler, L. Yoon, H. Kim, & S. Stich. in preparation Distinct cultural pathways to moral parochialism: Evidence from Los Angeles and Seoul. *Manuscript in preparation*.
